# Beneficial Effects of Gracillin From *Rhizoma Paridis* Against Gastric Carcinoma via the Potential TIPE2-Mediated Induction of Endogenous Apoptosis and Inhibition of Migration in BGC823 Cells

**DOI:** 10.3389/fphar.2021.669199

**Published:** 2021-09-24

**Authors:** Wenming Liu, Yanting Wang, Junjie Chen, Zhenhe Lin, Mengjie Lin, Xiantong Lin, Yanyun Fan

**Affiliations:** ^1^ Department of Gastroenterology, Zhongshan Hospital, Xiamen University, Xiamen, China; ^2^ Department of Pathology, Zhongshan Hospital, Xiamen University, Xiamen, China; ^3^ Department of Thoracic and Cardiovascular Surgery, The Third Hospital of Xiamen, Xiamen, China

**Keywords:** TIPE2, gracillin, rhizoma paridis, gastric carcinoma, BGC-823

## Abstract

Tumor necrosis factor-α inducible protein-8 (TIPE2), initially recognized as a negative immune regulator, exerts an important role in suppressing the progression of numerous cancers. In our previous investigation, we found that TIPE2 expression displayed a decrease or absence in gastric tumor tissue, and the overexpression of TIPE2 suppressed the growth of gastric cancer tumors and cells, demonstrating that TIPE2 could be a potential medicinal target for gastric cancer treatment. However, it’s seldomly reported that several medicinal agents or candidates targeted TIPE2 for treating diseases, including gastric cancer. To identify the candidate targeting TIPE2 to fight against gastric cancer, several extractions from traditional natural medicinal plants with anti-tumor functions were employed to screen the active compounds according to bioassay-guided isolation. Interestingly, gracillin, a component from the ethyl acetate extraction of *Rhizoma Paridis*, was identified to induce the expression of TIPE2 and inhibit the cell proliferation in gastric cancer BGC-823 cells. Furthermore, the underlying mechanisms that restrain gastric cancer were evaluated by clone formation, EdU staining, flow cytometry, and other assays. Meanwhile, the role of TIPE2 in the anti-tumor effect of gracillin was elucidated via the use of siTIPE2 RNA. It was determined that gracillin could fight against gastric cancer cells by inhibiting the cell proliferation participated by the PI3K/AKT pathway and cell cycle arrest, suppressing the EMT pathway-regulating cell migration, and inducing bcl2-associated mitochondrial apoptosis. Additionally, TIPE2 maybe contribute to the benefits of gracillin. These results of the present study are an important step toward the medicinal development of gracillin, and are also of use in understanding the effect of TIPE2 as a potential tumor target.

## Introduction

Gastric cancer has become the fourth most common incidence and the second most common cause of death globally, as such is a serious threat to human health and happiness (Rui et al., 2019; Elizabeth et al., 2020; Tony et al., 2020; Yoshiki, 2020; Hyuna et al., 2021). Therapeutic strategies for treating gastric cancer have been challenged by the complex and controversial causes of gastric cancer development (Antonia and Wagner, 2016; Helge and Reidar, 2018; Camilla and Giuseppe, 2020; Nalinie et al., 2020). For several decades, chemotherapy has remained one of the most commonly used therapies following surgical treatment to fight against gastric cancer. However, the traditional chemotherapeutical agents, such as 5-fluorouracil, cyclophosphamide, and other broad-spectrum anti-tumor agents, presented serious toxic side effects, such as weight loss, hair loss, and anorexia, during the treatment of gastric cancer. This has been attributed to their lack of selectivity for healthy and tumor cells (Mary et al., 2020; Yong et al., 2021). To improve the selectivity of chemotherapeutical agents, drugs targeting proteins specifically expressed in tumor tissue or selectively-mediated tumor have aroused great interest, and a few target drugs have also been effectively developed to treat liver cancer, lung cancer, and gastric cancer in the clinic (G Schinzari et al., 2014).

Tumor necrosis factor-α inducible protein-8 (TNFAIP8L2, also called TIPE2) is a negative immune mediator that was discovered in 2008 (Eric et al., 2008; Honghong et al., 2008). The emerging evidence shows that TIPE2 could inhibit gastric carcinoma growth and metastasis (Qian et al., 2015; Jie et al., 2016; Ganesan et al., 2018). Our previous research suggested that TIPE2 is missing or has low expression in gastric carcinoma but not in normal gastric mucosa (Wenming et al., 2018; Zhenhe et al., 2018). Furthermore, the overexpression of TIPE2 could suppress the proliferation and migration of gastric cancer BGC823 cells. These data demonstrate that TIPE2 could be a novel potential therapeutic target for gastric cancer treatment. However, candidates targeting TIPE2 to restrain gastric carcinoma are as yet seldom reported. Therefore, exploring TIPE2’s mediator would be an important and significant strategy in the development of new anti-tumor medicine for gastric cancer.

Natural products, especially those found in various traditional medicine systems such as Traditional Chinese Medicine (TCM), have served as folk medicinal agents to regulate immune, inflammation, metabolism, and other physiologies for thousands of years (Daniel et al., 2012; Tai et al., 2019). The medicine of natural products was wildly applied to relieve all kinds of diseases including cancer (Amit and Vikas, 2019; Min et al., 2019; Tohid et al., 2019; Xuanmei et al., 2019). Excitingly, Lianhuaqingwen, (TCM) formula consisting of multiple natural herbs such as fructus forsythiae, Lonicera japonica Thunb, and Houttuynia cordata Thunb have also contributed to controlling the 2019 novel SARS-CoV-2 global pandemic (Li et al., 2020; Ming et al., 2021). These natural products had crucial roles in maintaining individual’s health and defeating disease, in both history and present. However, its complicated ingredients with numerous different yet remarkably similar structural molecules hindered the modern clinical application. In the last hundred years, these pure and simple pharmaceutical agents are slipping into the mainstream of clinical drugs, rather than natural product mixtures (Emma and Margaret, 2019). Fortunately, the majority of these pure medicinal compounds were sourced or derived from natural product components. From 1981 to 2014, 65% of all single pharmaceutical agents approved by the American Food and Drug Administration (FDA) came from natural product components and their derivatives. Furthermore, 75% of the anti-tumor agents came from natural products and their derivatives (David and Gordon, 2016). In the future, natural products will remain a valuable source of novel medicine agents, and identifying potential lead molecules from natural product mixtures is still an important strategy of drug discovery.

In this study, we aimed to identify a potential TIPE2 candidate for restraining gastric carcinoma from a traditional natural herb, and to clarify the mechanism by which the candidate suppresses gastric carcinoma via TIPE2. The present research started with screening active compounds for inhibiting the proliferation of gastric cancer cell BGC-823 and regulating TIPE2 protein expression from the fractions of four medicinal plants *Curcuma longa L., Tripterygium Wilfordii, Rhizoma Paridis,* and *Reynoutria japonica Houtt*. Following screening the active fractions from the medicinal plant extracts and identifying the potential candidate from the active fractions via bioassay-guided isolation, a TIPE2 mediator named gracillin from *Rhizoma Paridis* was discovered to have potential in suppressing gastric cancer cell proliferation. Furthermore, we assessed the mechanism by which gracillin suppressed gastric carcinoma, and found that gracillin could induce cellular apoptosis and inhibit migration via TIPE2-mediated endogenous apoptosis and EMT pathway in BGC-823. The results provided a theoretical basis for developing drugs to treat gastric cancer by targeting TIPE2.

## Materials and Methods

### Preparation of Medicinal Plants Extraction and the Identification of Active Compounds

The medicinal plants *Curcuma longa L., Tripterygium Wilfordii, Rhizoma Paridis*, and *Reynoutria japonica Houtt* were purchased from the Traditional Chinese Medicine Trade Center of Bozhou in Anhui Province, China. These plants were first crushed into a coarse powder and soaked for 2 h in six volumes of 60% ethanol (volume/weight) to extract by reflux. The extraction solution was filtered, and the residue was subjected to this process two more times. The filtered solutions were merged and concentrated to obtain the total extraction using rotary evaporation (EYELA, Japan). The total extraction was suspended in water and extracted with petroleum ether, chloroform, and ethyl acetate (Sinopharm, Beijing, China) to obtain the fractions of PE, C, and EA. After a bio-activities test, the active fractions were separated using preparative high-performance liquid chromatography with a flow phase of 40–55% acetonitrile (Merck, Germany) and the active compounds were identified by mass spectroscopy.

### Cell Culture

Human gastric tumor BGC-823 cells (Chinese Academy of Medical Sciences, Shanghai, China) were cultured in an RPMI-1640 medium (Hyclone, Utah, United States) containing 10% FBS (Gibco, Waltham, United States) and 1% bi-antibiotic of penicillin and streptomycin (Hyclone, Utah, United States) in an atmosphere of 5% CO_2_ and 37°C (Thermofisher, MA, United States). Before the bio-assays of agents were performed, BGC-823 cells were seeded into the preassigned culture plates and administrated with the different plant extractions or gracillin for a corresponding time. Then the cells were harvested and the detection was carried out according to standard protocol.

### MTT Assay

Cell proliferation was evaluated using an MTT assay. Briefly, BGC-823 cells were seeded into a 96-well plate at a density of 10,000 cells/well and cultured for 12 h. Then the seeded cells were administrated with plant extractions or gracillin and cultured for 24/48 h. An MTT solution with 5 mg/ml (Solarbio, Beijing, China) was added to the well and incubated for another 3 h. After incubation, the medium was removed, the cells were washed twice with PBS, and 100 μL DMSO (Solarbio, Beijing, China) was added to each well to dissolving the formazan. Finally, the absorbance was read at 490 nm using a microplate reader (Thermofisher, MA, United States) and the proliferation ratio was calculated as follows:

### Western Blot

BGC-823 cells were seeded into a 6-well plate with 60% confluence and maintained in a CO_2_ incubator for 12 h. Then the seeded cells were administrated with different doses of gracillin for another 12 h. The treated cells were harvested, lysed in a RIPA buffer (Solarbio, Beijing, China) on ice with a vortex of once per 5 min for 30 min, and centrifuged at 12,000 rpm and 4°C. The supernatants were collected, the total protein concentration was evaluated by BCA assay kit (Thermofisher, MA, United States), and boiled using dry baths (Yiheng, Hangzhou, China) to denature the protein. A sample of 20 μg of prepared protein was subjected to 8–12% SDS-PAGE to separate the total protein and then transferred to a PVDF membrane (Millipore, MA, United States). The transferred PVDF membrane was immersed in 5% defatted milk at room temperature for 1 h to block, incubated overnight at 4°C with the primary antibodies of TIPE2, AKT, p-AKT, CDK1, p-CDK1, cyclin B1, p21, E-cadherin, N-cadherin, vimentin, cleaved PARP, bcl2, cleaved caspase3, and cleaved caspase9 (CST, United States), then incubated with a secondary antibody (Proteinteck, Wuhan, China) at room temperature for 1 h. Finally, ECL chemiluminescent solution was added to the PVDF membranes (Pierce, United States) for imaging.

### EdU Staining

The cell proliferation was evaluated using EdU staining. Briefly, BGC-823 cells and BGC823/TIPE2^−/−^ cells were seeded into a 12-well plate with 30% confluence and maintained in a CO_2_ incubator for 12 h. Then the seeded cells were administrated with 5 μM gracillin and cultured. Later, the cells were added with EdU solution to stain for 2 h, washed with PBS, and imaged with a microscope (Olympus, Japan).

### Colony Formation

The cell proliferation was also assessed using colony formation. Briefly, BGC-823 cells and BGC823/TIPE2^−/−^ cells were seeded into a 12-well plate with 5,000 cells per well and maintained in a CO_2_ incubator for 12 h. Then the seeded cells were administrated with 5 μM gracillin and cultured for 7 days. Later, the cells were washed with PBS and fixed with 4% paraformaldehyde and then stained with gimsa solution for 30 min and imaged.

### Cell Cycle Distribution Analysis

BGC-823 cells and BGC823/TIPE2^−/−^ cells were seeded into a 6-well plate with 50% confluence and maintained in a normal medium for 12 h, then replaced with an FBS-free medium to culture for another 12 h. After starvation culture, the cells were treated with 3 μM gracillin for 24 h. Then the treated cells were collected, fixed with pre-cooled 70% ethanol overnight at 4°C, and stained with 5 μg/ml propidium iodide (Solarbio, Beijing, China) at room temperature for 20 min. Finally, the stained cells were detected using the flow cytometer (Beckman, United States).

### Scratch Assay

The cell migration was assessed using a scratch assay. Briefly, BGC-823 cells and BGC823/TIPE2^−/−^ cells were seeded into a 6-well plate with 60% confluence and cultured for 12 h. Then the seeded cells were scratched with a white pipette tip to form a cell-free area and administrated with 3 μM gracillin for 48 h. The cell-free area was imaged and its width was quantified using Image J software.

### Flow Cytometer for Cell Apoptosis

The cell apoptosis was examined by dual-staining with FITC-Annexin V and PI. Briefly, BGC-823 cells and BGC823/TIPE2^−/−^ cells were seeded into a 6-well plate with 60% confluence and cultured for 12 h. Then the seeded cells were administrated with 3 μM gracillin for 24 h. The cells were digested with a 0.25% trypsin solution and transferred to a 1.5 ml EP tube, then suspended in 500 μL binding buffer, and dyed with 5 μLAnnexin V-FITC and 5 μL PI according to the manufacturer’s manual of FITC-Annexin apoptosis detection kit (BD, United States). The stained cells were detected using a flow cytometer (Beckman, United States).

### Animal Experiment

Male nude mice aged 6 weeks were purchased from the Xiamen University Laboratory Animal Center. The mice were raised in pathogen-free conditions with 12 h light/12 h dark cycles for a week, then randomly divided into two groups, the control group and the gracillin administration group, and subcutaneously inoculated with BGC-823 gastric cancer cells of 2×10^6^ cells per mice. The inoculated mice were maintained for another week, then injected with gracillin or normal saline once every other day for 21 days. During this period, the tumor size and body weight were measured once every 3 days. On the 21st day, the mice were sacrificed and their tumors were collected for immunohistochemistry and western blotting test.

### Hematoxylin and Eosin Staining and Immunohistochemistry Staining

Tumor tissue specimens were fixed in 4% paraformaldehyde for 1 h, then soaked in wax and sectioned. The sections were firstly dewaxed via incubation at 60°C for 1 h, soaked twice in xylene for 5 min, then in 100, 100, 95, 80% alcohol for 1 min each. In HE staining, the dewaxed sections were stained with hematoxylin for 6 min and eosin for 2 min, then washed with distilled water, dehydrated with gradient alcohol (95, 95, 100, and 100%) for 1 min each, vitrified with xylene twice, and sealed with neutral gum. In IHC staining, the dewaxed sections were immersed in a citrate buffer, washed with 0.01 M PBS, and cooled for antigen repair. Then the sections were incubated with the primary antibody ki67 overnight and a secondary antibody for 1 h. Finally, the specimens were stained with hematoxylin, dehydrated, vitrified, and sealed.

### Statistical Analysis

Statistical analysis was carried out using SPSS software. The data were displayed as mean± (SEM). One-way ANOVA was employed to compare the differences between groups. *p* < 0.05 was considered statistically significant.

## Results

### The Main Components Analysis of Active Fractions From Natural Plants

**FIGURE 1:**
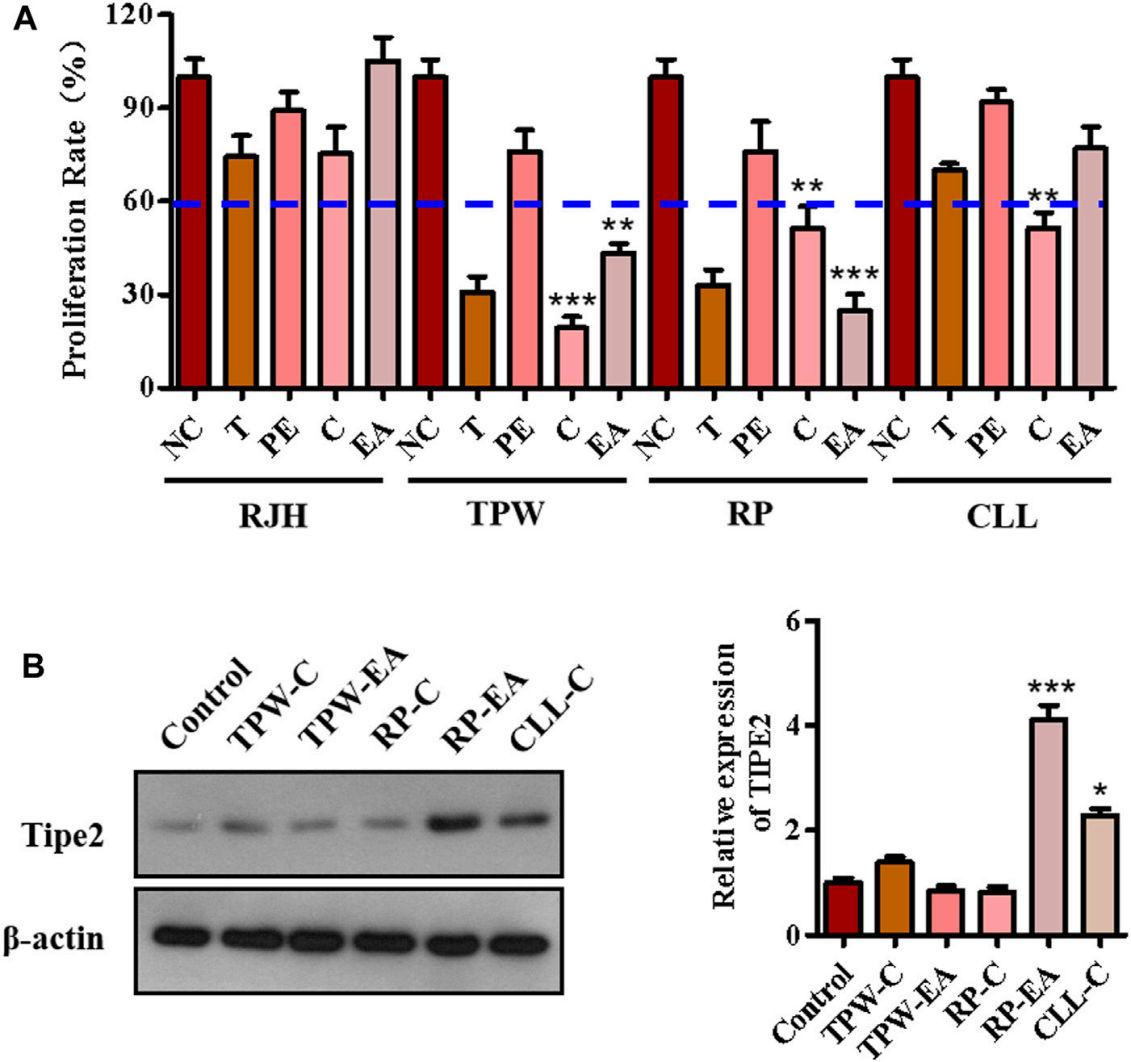
The components analysis of active fractions from medicinal plants. **(A)** The effect of medicinal plants on cell proliferation in BGC823 cells. 50 μg/ml extractions of the plants were used to treat BGC823 cells for 48 h. **(B)** The effects of active fractions on the expression of TIPE2. 50 μg/ml active fractions were used to treat the BGC823 cells for 12 h. The values in the histogram are displayed as mean ± SD; 0.01 < *p* < 0.05 (*) represents a significant difference, and *p* < 0.001 (***) represents an extremely significant difference to the normal control.

The anti-proliferation effect of fractions from four traditional natural plants, *Reynoutria japonica Houtt (RJH), Tripterygium Wilfordii (TPW), Rhizoma Paridis (RP),* and *Curcuma longa L. (CLL),* were first examined by MTT assay to screen active factions. As shown in Figure 1A, five fractions (chloroform extraction and ethyl acetate extraction from *Tripterygium Wilfordii and Rhizoma Paridis* (TPW-C, TPW-EA, RP-C, and RP-EA) and chloroform extraction from *Curcuma longa L.*(CLL-C)) showed a proliferation inhibitory ratio of more than 40% compared with the control group (Figure 1A). In these active fractions, RP-EA exhibited an evident induction of TIPE2 protein expression in comparison with the control group (Figure 1B), implying that some active compounds with the potential to induce TIPE2 expression and inhibit gastric tumor cell proliferation could exist in ethyl acetate extraction from *Rhizoma Paridis*. To identify the active compounds, high-performance liquid chromatography (HPLC) and mass spectroscopy were employed to separate and characterize the main components of RP-EA extraction. Five obvious peaks detected by HPLC chromatography were identified as polyphyllin II (PPL-II), dioscin, gracillin, polyphyllin I (PPL-I), and polyphyllin VI (PPL-VI) using mass spectroscopy (Supplementary Figures S1–S6).

### Identifying Gracillin as a Tumor Necrosis Factor-α Inducible Protein-8 Inducer With Potential Suppressing Cell Proliferation in Gastric Cancer BGC823 Cells

**FIGURE 2:**
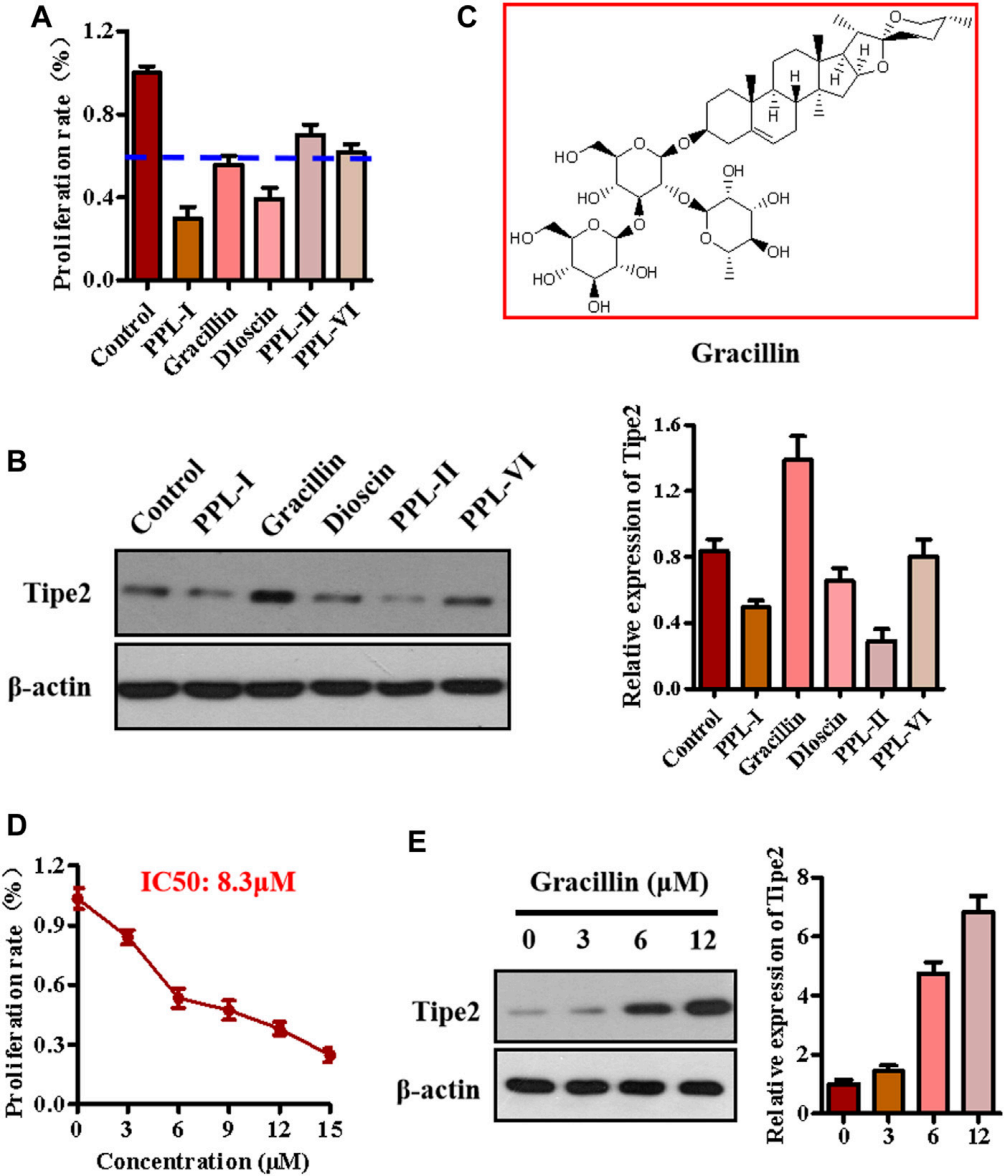
The effect of gracillin on TIPE2 expression and cell proliferation in BGC-823 cells. **(A)** The effect of five active compounds on cell proliferation. 5 μM active compounds were used to treat the BGC823 cells for 48 h. **(B)** The effect of five active compounds on TIPE2 expression. 5 μM active compounds were used to treat the BGC823 cells for 12 h. **(C)** The structure of gracillin. **(D)** The effect of gracillin at different concentrations on cell proliferation. The different concentrations of gracillin with 3, 6, 9, 12, 15 μM were used to treat the BGC823 cells for 12 h. **(E)** The effect of gracillin with the indicated concentrations for 12 h on TIPE2 expression. The values of the histogram are displayed as mean ± SD.

The above experiments identified five main components from the chloroform extraction of *Rhizoma Paridis.* We next screened the active compound with the highest potential for regulating TIPE2 and gastric carcinoma from these main components via MTT and immunoblotting. MTT assay showed that polyphyllin I, gracillin, and dioscin exhibited lower cellular proliferation ratio (lower than 60 versus 100% of the control group, Figure 2A), meanwhile, immunoblotting indicated that gracillin had an obvious induction of TIPE2 expression in BGC823 cells (Figure 2B), demonstrating that gracillin could be a TIPE2 inducer suppressing gastric tumor cell proliferation. The structure of gracillin was shown in Figure 2C. In addition, gracillin concentration-dependently inhibited the gastric tumor BGC823 cell proliferation with IC50 value of 8.3 μM in the range of 0–15 μM (Figure 2D) and gastric tumor SGC7901 cell proliferation with IC50 value of 8.9 μM in the range of 0–15 Μm (Supplementary Figure S7). TIPE2 expression tests also showed that gracillin exhibited a concentration-dependent TIPE2 induction in 3, 6, and 12 μM (Figure 2E and Supplementary Figure S10).

### Establishment of Tumor Necrosis Factor-α Inducible Protein-8-Silence Cell Line BGC823/Tumor Necrosis Factor-α Inducible Protein-8^−/−^

**FIGURE 3:**
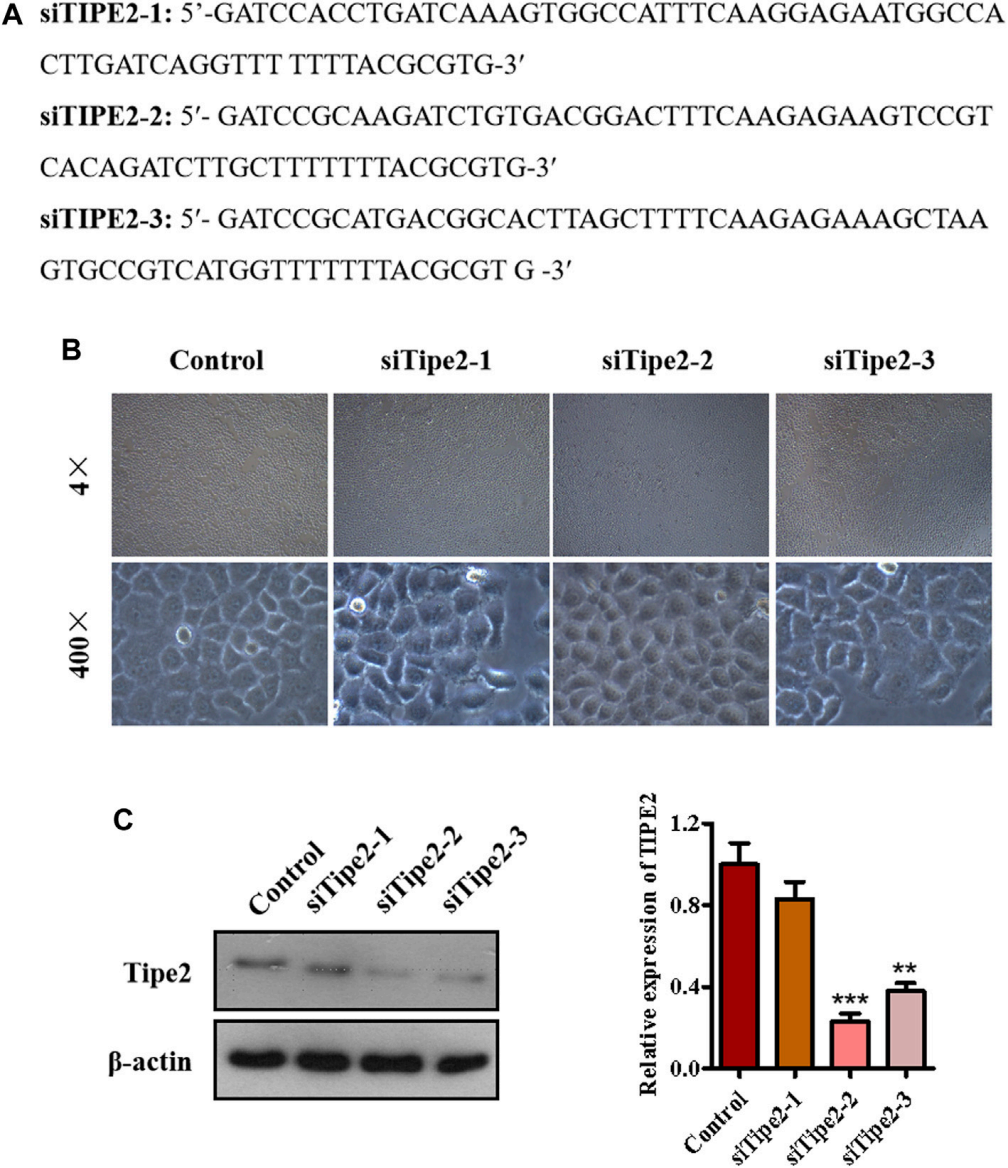
The establishment of TIPE2-silence cell line BGC823/TIPE2−/−. **(A)** The sequence of siTIPE2 RNA. **(B)** The effect of siTIPE2 RNA on BGC-823 cells. **(C)** The effect of siTIPE2 RNA on TIPE2 expression. The values of the histogram are displayed as mean ± SD, 0.001 < *p* < 0.01 (**) represented for very significant difference and *p* < 0.001 (***) represents an extremely significant difference to the normal control.

To explore the role of TIPE2 in gracillin suppression of gastric tumors, we synthesized three fragments of TIPE2 siRNA sequence, which were respectively siTIPE2-1, siTIPE2-2, and siTIPE2-3 (Figure 3A). The fragments were transfected into BGC823 gastric tumor cells and Figure 3B shows the BGC823 and BGC823/TIPE2^−/−^ cellular images of TIPE2 siRNAs transfection for 36 h. The transfection efficiency was evaluated by immunoblotting. As displayed in Figure 3C, siTIPE2-2 and siTIPE2-3 inhibited the expression of TIPE2 in BGC823. Furthermore, siTIPE2-2 was more efficient compared with siTIPE2-3. Therefore, we choose siTIPE2-2 as a tool for suppressing TIPE2 expression to illustrate the role of TIPE2 in gracillin suppression of gastric tumors.

### Effects of Gracillin on Cellular Proliferation via Tumor Necrosis Factor-α Inducible Protein-8 in BGC823 Cells

**FIGURE 4:**
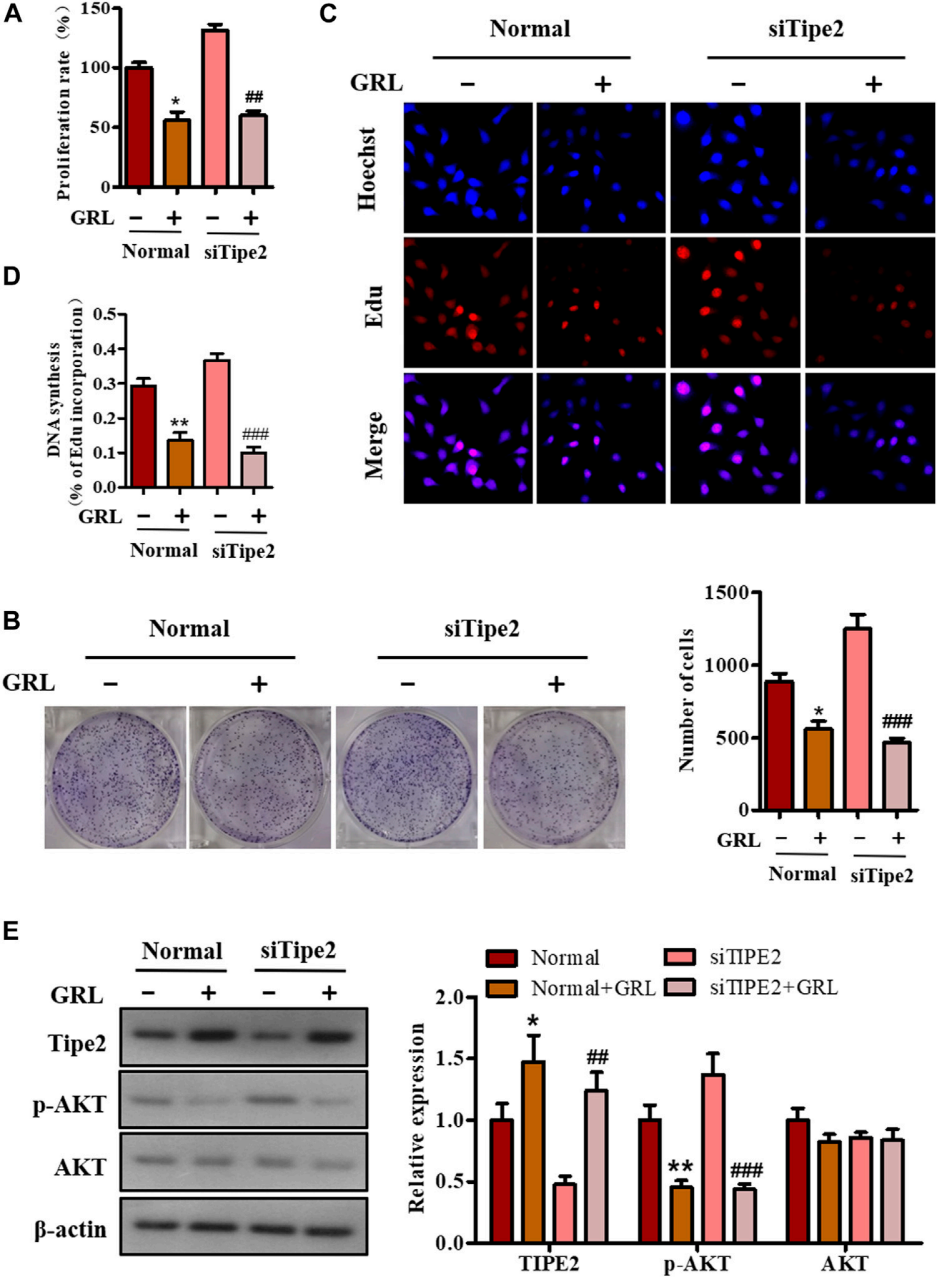
The role of TIPE2 in cell proliferation mediated by gracillin in BGC823 cells. **(A)** MTT assay for cell proliferation by TIPE2 and gracillin. 5 μM gracillin was used to treat the BGC823 cells and TIPE2 siRNA BGC823 cells for 48 h. **(B)** Colony formation for cell proliferation by TIPE2 and gracillin. 5 μM gracillin was used to treat the BGC823 cells and TIPE2 siRNA BGC823 cells for 7 days. **(C)** EdU staining for cell proliferation by TIPE2 and gracillin. 5 μM gracillin was used to treat the BGC823 cells and TIPE2 siRNA BGC823 cells for 12 h. **(D)** The quantitative analysis for EdU staining. **(E)** Western blot for the expression of TIPE2, AKT, and p-AKT. The values of the histogram are displayed as mean ± SD. 0.01 < *p* < 0.05 (*) represents a significant difference and 0.001 < *p* < 0.01 (**) represents a very significant difference to the normal control; 0.001 < *p* < 0.01 (^##^) represents a very significant difference and *p* < 0.001 (^###^) represents an extremely significant difference to the TIPE2 siRNA control.

To confirm whether TIPE2 participated in gracillin inhibition of gastric tumor cellular growth, the BGC823 and BGC823/TIPE2^−/−^ cellular viability in the administration of gracillin (5 μM) were first examined using MTT assay. As seen in Figure 4A, 5 μM of gracillin inhibited cell proliferation from 131.6 to 60.0% in BGC823/TIPE2^−/−^ cells, and from 100 to 56.0% in BGC823 cells, demonstrating that the proliferation inhibitory ratio of gracillin with siTIPE2 RNA treatment is higher than that without siTIPE2 RNA treatment (54.4 vs 44.0%) in BGC823 cells. Plate clone formation assay showed that the cellular clone number in BGC823/TIPE2^−/−^ cells subjected to gracillin decreased to 466 from 1,247 in the control group, and in BGC823 cells subjected to gracillin decreased to 557 from 885 (Figure 4B), indicating that gracillin more markedly suppressed the cellular clone formation in BGC823/TIPE2^−/−^ cells compared with that in BGC823 cell. EdU-incorporation data revealed the consistent effect that the proportion of incorporated EdU with gracillin treatment presented a more obvious reduction in BGC823/TIPE2^−/−^ cells compared with BGC823 cells (Figures 4C,D). Collectively, these results suggested that TIPE2 may participate in gracillin inhibition of cell proliferation in BGC823.

PI3K/AKT is an important signaling pathway involved with cell proliferation and phosphorylation of AKT is an essential molecular event in the process of PI3K/AKT activation. To further confirm whether gracillin inhibited gastric tumor cell proliferation via TIPE2, we conducted western blot assay to detect the AKT phosphorylation in the supplement of gracillin and siTIPE2 RNA in BGC823 cells. The data showed that gracillin more evidently promoted the induction of TIPE2 in BGC823/TIPE2^−/−^ than in BGC823, meanwhile, the inhibition effect of AKT phosphorylation by gracillin is stronger in BGC823/TIPE2^−/−^ than that in BGC823 (Figure 4E). Thus, TIPE2 maybe contribute to the anti-proliferation effect by gracillin.

### Effects of Gracillin on Cell Cycle Arrest via Tumor Necrosis Factor-α Inducible Protein-8 in BGC823 Cells

**FIGURE 5:**
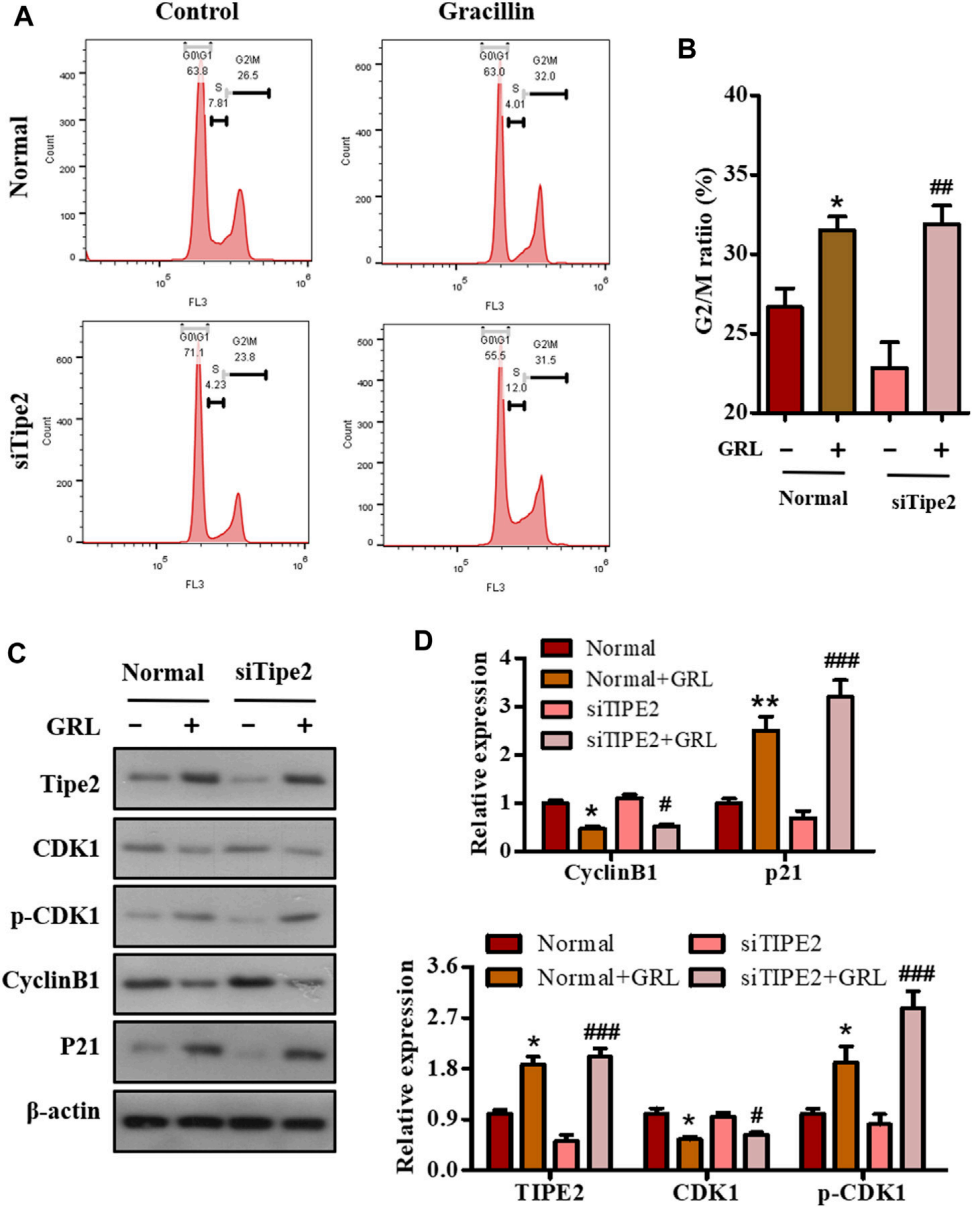
The role of TIPE2 in the cell cycle distribution mediated by gracillin in BGC823 cells. **(A)** Flow cytometry for cell cycle distribution by TIPE2 and gracillin. BGC823 cells and TIPE2 siRNA BGC823 cells were maintained with FBS-free medium for 36 h, then treated with 3 μM gracillin for another 24 h. **(B)** The quantitative analysis of cell cycle distribution by TIPE2 and gracillin. **(C)** Western blot for the expression of TIPE2, CDK1, p-CKD1, cyclin B1, and p21. **(D)** The quantitative analysis of protein expression. The values of the histogram were displayed as mean ± SD. 0.01 < *p* < 0.05 (*) represents a significant difference and 0.001 < *p* < 0.01 (**) represents a very significant difference to the normal control; 0.01 < *p* < 0.05 (^#^) represents a significant difference, 0.001 < *p* < 0.01 (^##^) represents a very significant difference and *p* < 0.001 (^###^) represents an extremely significant difference to the TIPE2 siRNA control.

Deregulation of the cell cycle results in the unlimited proliferation of tumor cells including gastric carcinoma cell BGC823, and controlling the deregulated cell cycle would be a crucial strategy to fight against tumors. To evaluate the association of the cell cycle with cell proliferation mediated by gracillin, BGC823/TIPE2^−/−^ and BGC823 cells with or without gracillin were stained by PI, and the cell cycle distribution was analyzed using flow cytometry. The histogram from flow analysis indicated that gracillin induced the increase of G2/M phase ratio in BGC823 cells, meanwhile, the induction of the G2/M phase ratio by gracilllin was more evident in BGC823/TIPE2^−/−^ cells (Figures 5A,B). Moreover, the expression of G2/M phase-associated proteins CDK1, p-CDK1, cyclin B1, and p21 in the supplement of gracillin were detected by western blot. The band graph revealed that gracillin induced the upregulation of TIPE2, p-CDK1, and p21 and downregulation of CDK1 and cyclin B1 in BGC823 cells, meanwhile, these change trends of protein expressions mediated by gracillin were more evident in BGC823/TIPE2^−/−^ cells (Figures 5C,D). These results demonstrated that TIPE2 maybe contribute to the cell cycle G2/M arrest effect induced by gracillin in BGC823 cells, which was consistent with our previous report that TIPE2 blocks the cell cycle G2/M phase (Zhenhe et al., 2018).

### Effects of Gracillin on Cellular Migration via Tumor Necrosis Factor-α Inducible Protein-8 in BGC823 Cells

**FIGURE 6:**
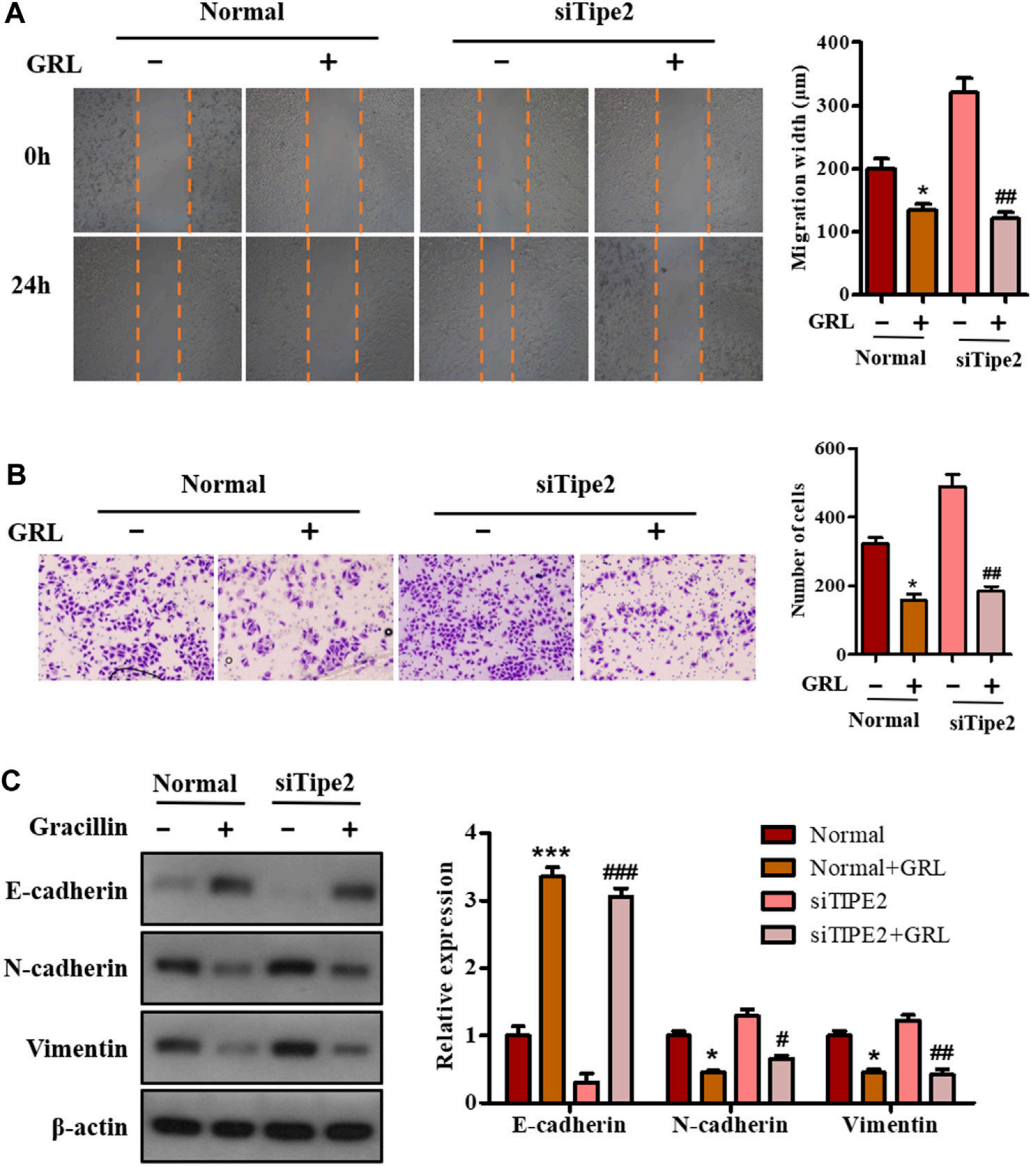
The role of TIPE2 in the cell migration mediated by gracillin in BGC823 cells. **(A)** Scratch assay for cell migration by TIPE2 and gracillin. BGC823 cells and TIPE2 siRNA BGC823 cells were treated with 3 μM gracillin for 24 h. **(B)** Transwell assay for cell migration by TIPE2 and gracillin. **(C)** Western blot for the expression of E-cadherin, N-cadherin, and vimentin. 0.01 < *p* < 0.05 (*) represents a significant difference and *p* < 0.001 (***) represents an extremely significant difference to the normal control; 0.01 < *p* < 0.05 (^#^) represents a significant difference, 0.001 < *p* < 0.01 (^##^) represents a very significant difference and *p* < 0.001 (^###^) represents an extremely significant difference to the TIPE2 siRNA control.

To investigate the effect of gracillin on gastric cell migration and the role of TIPE2 in this effect, scratch-wound assay and transwell assay were performed in BGC823/TIPE2^−/−^ and BGC823 cells. As seen in Figure 6A, the migration width of BGC823 cells in the supplement of gracillin for 24 h decreased by 32.2% (from 199 to 135 μm), and BGC823/TIPE2^−/−^ cells displayed a higher fall of migration width of 62.2% (from 320 to 121 μm) induced by gracillin. The data from the transwell assay also revealed that gracillin inhibited the cell migration, which displayed a fall of cell migrations from 321 to 157 in BGC823 cells, while a larger decrease was observed in BGC823/TIPE2^−/−^ cell. Additionally, the expression of EMT pathway-related proteins was detected by western blot to study the molecular mechanism by which TIPE2 mediates cell migration in gracillin. Figure 6C shows that gracillin induced the upregulation of E-cadherin and downregulation of N-cadherin and vimentin in BGC823 cells, and these trends maintain a similar level in BGC823/TIPE2^−/−^ cells. Taken together, these results demonstrate that gracillin could inhibit the cell migration via the EMT pathway in BGC823 cells and TIPE2 maybe contribute to the inhibitory effect of gracillin.

### Effects of Gracillin on Cellular Apoptosis via Tumor Necrosis Factor-α Inducible Protein-8 in BGC823 Cells

**FIGURE 7:**
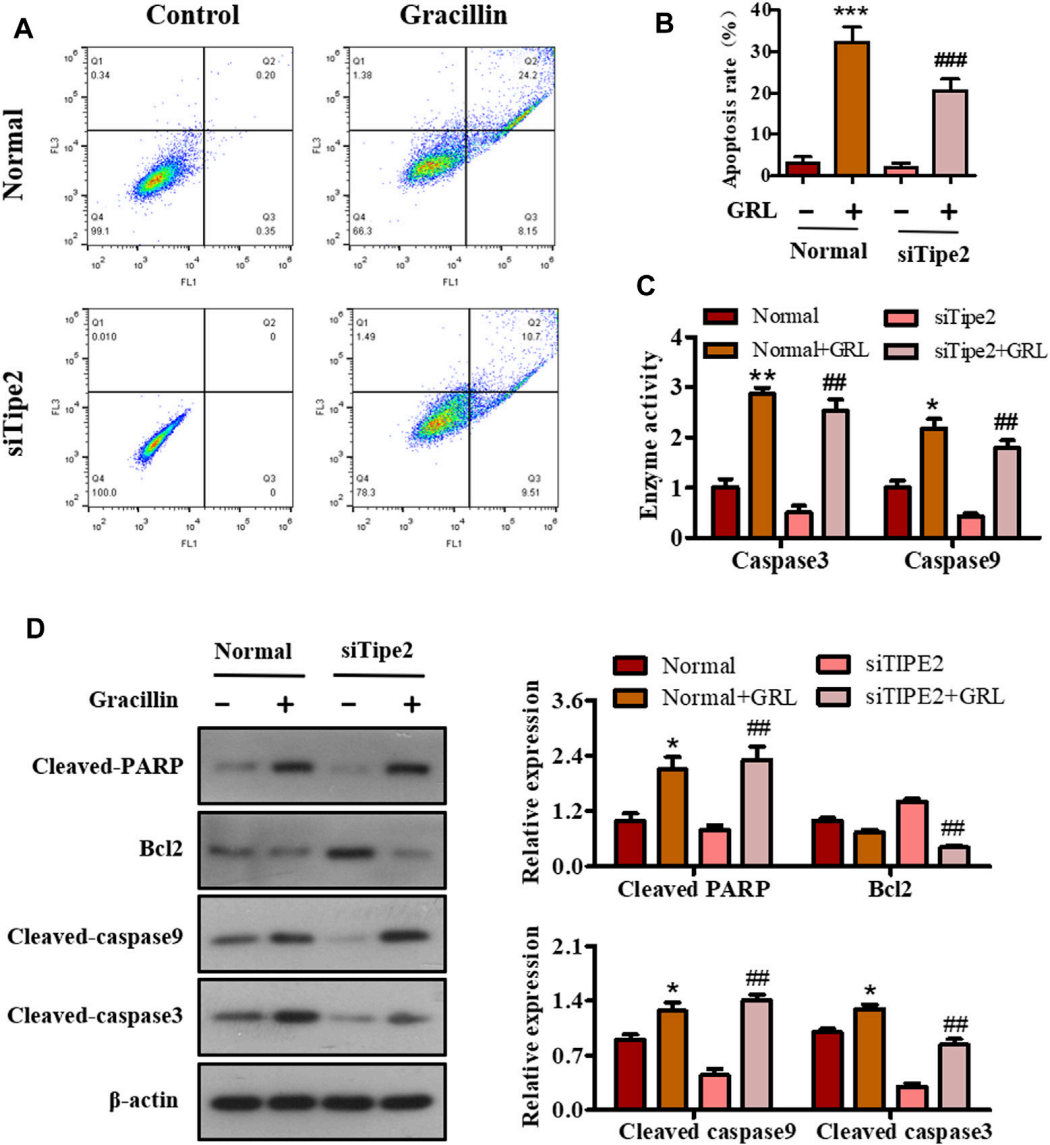
The role of TIPE2 in the cell apoptosis mediated by gracillin in BGC823 cells. **(A)** Flow cytometer for cell apoptosis by TIPE2 and gracillin. BGC823 cells and TIPE2 siRNA BGC823 cells were treated with 3 μM gracillin for 24 h. **(B)** The quantitative analysis of cell apoptosis. **(C)** Elisa assay for the enzyme activities of caspase three and caspase 9. **(D)** Western blot for the expression of cleaved PARP, bcl2, cleaved caspase 9, and cleaved caspase 3. 0.01 < *p* < 0.05 (*) represents a significant difference, 0.001 < *p* < 0.001 (**) represents a very significant difference and *p* < 0.001 (***) represents an extremely significant difference to the normal control; 0.01 < *p* < 0.05 (^#^) represents a significant difference, 0.001 < *p* < 0.01 (^##^) represents a very significant difference and *p* < 0.001 (^###^) represents an extremely significant difference to the TIPE2 siRNA control.

Sustained proliferation and resistance to cell apoptosis are two major hallmarks of cancer. The above-mentioned data have shown that gracillin could fight against gastric cancer via TIPE2-regulation of cell proliferation. We performed flow cytometry, enzyme activity tests, and western blot assay to study the apoptotic effect of gracillin via TIPE2 in BGC823 cells. Scatter diagrams flow cytometry showed that the apoptosis ratio of BGC823 cells supplied with gracillin increased from 3.11 to 32.24% while BGC823/TIPE2^−/−^ cells displayed a similar change trend (Figures 7A,B). Enzyme activity tests (Figure 7C) revealed that gracillin induced the enzyme activities of caspase three and nine in BGC823 cells and BGC823/TIPE2^−/−^ cells, and that caspase9 activity displayed a more obvious induction in BGC823/TIPE2^−/−^ cells than that in BGC823 cells. Western blot (Figure 7D) showed that gracillin induced the cleavage of PARP, caspase3, and caspase9, and the inhibition of bcl-2 expression in BGC823 cells, furthermore, the regulation by gracillin in BGC823/TIPE2^−/−^ cells were more statistically significant. These results demonstrate that TIPE2 maybe contribute to the pro-apoptosis effect of gracillin in BGC823 cells.

### Effects of Gracillin on Gastric Tumor and Tumor Necrosis Factor-α Inducible Protein-8 Expression *in vivo*

**FIGURE 8:**
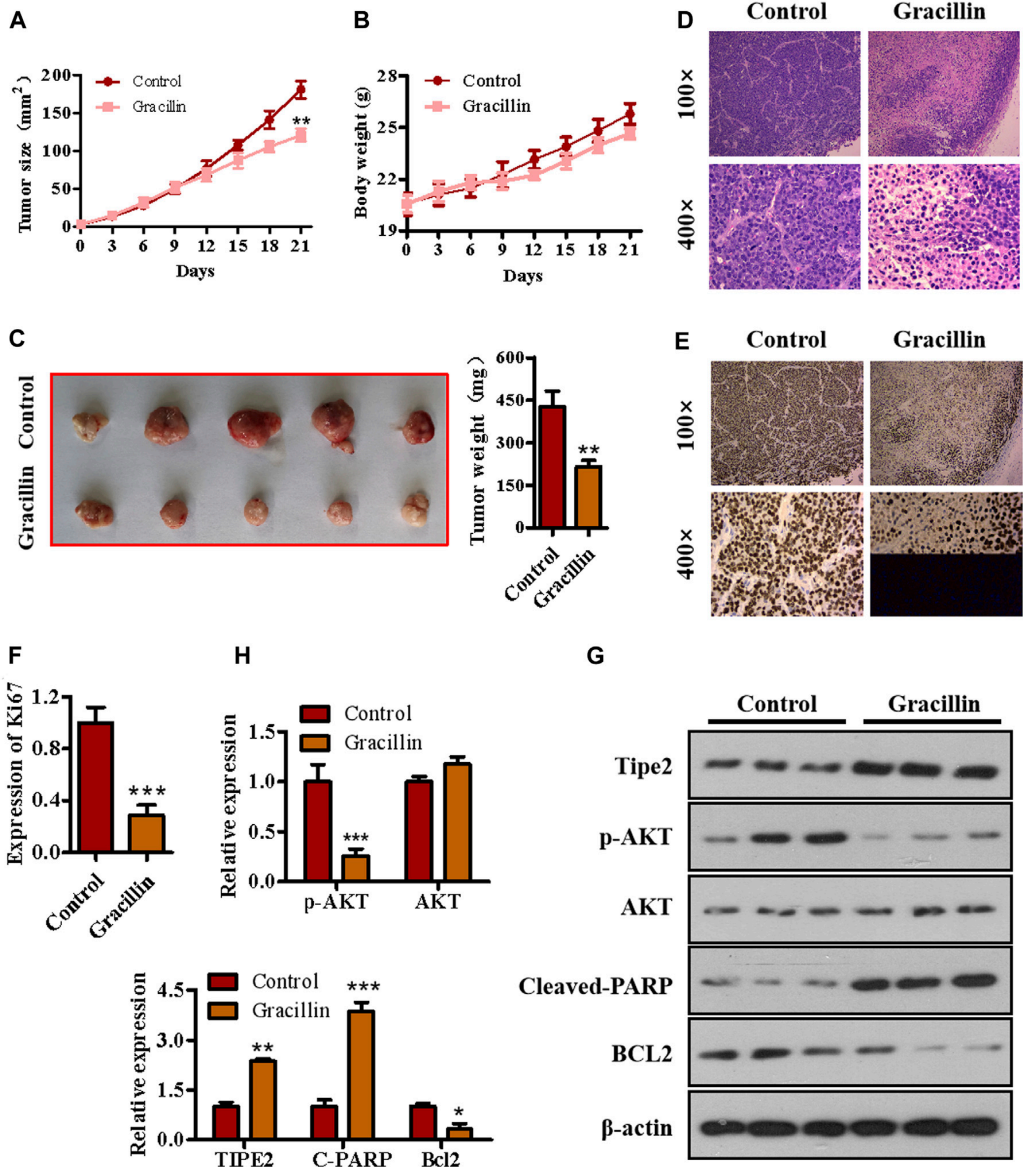
The effect of gracillin on gastric tumor and TIPE2 expression *in vivo*. **(A)** The growth curve of tumors. **(B)** The curve of body weight. **(C)** The tumor images. **(D)** HE stain for tumor pathology. **(E)** IHC assay for ki67 expression in tumors. **(F)** The quantitative analysis of ki67 expression. **(G)** Western blot for the expression of TIPE2, p-AKT, AKT, cleaved PARP, and bcl2 in tumor tissue. **(H)** The quantitative analysis of protein expression as detected by western blot. 0.01 < *p* < 0.05 (*) represents a significant difference, 0.001 < *p* < 0.001 (**) represents a very significant difference and *p* < 0.001 (***) represents an extremely significant difference to the normal control.

To determine the anti-tumor effect of gracillin *in vivo*, a xenograft tumor model of nude mice bearing BGC823 cells was established and gracillin was administrated. As seen in Figure 8A, the tumor size of mice treated with gracillin began to fall compared to that of mice in the control group from the 12th day and reached a great significant difference at the end of the 21st day (**p < 0.01 compared to the control group). The curve of body weight showed that the mice that received gracillin had no statistical change of body weight compared with that in the control group. The tumor images and weight on the 21st day of gracillin administration revealed that gracillin suppressed the tumor size and tumor weight (**p < 0.01 compared to the control group, Figure 8C). The immunohistochemistry test revealed that gracillin greatly inhibited the expression of Ki67 in the mice tumor tissue (***p < 0.001 compared to the control group, Figures 8D–F). Additionally, western blot data (Figures 8G,H) showed that gracillin induced the cleavage of PARP and expression of TIPE2, and inhibited the phosphorylation of AKT and expression of bcl-2 in the tumor tissue of mice. Together, these results demonstrated that gracillin could inhibit tumors via TIPE2-mediation of proliferation and apoptosis.

## Discussion

Gastric cancer has been a serious threat to human life and health for several decades, which has promoted the identification of new therapeutic targets and the development of novel therapeutic agents for gastric cancer. TIPE2 protein was first identified only a few years ago, and since then a great deal of evidence has indicated that TIPE2 plays a crucial role in suppressing gastric cancer. Like other cancer cells, gastric cancer cells are characterized by sustained proliferative signaling, evasion of growth suppressors, activate invasion and metastasis, resistance to cell death, and so on (Douglas and Robert, 2011). The expression of TIPE2 could control these defects of gastric cancer cells. It had been verified that TIPE2 could suppress cell growth and proliferation by inducing the inhibition of the AKT and ERK1/2 pathways and promoting the p27-associated signal cascade in gastric cancer cells (Qian et al., 2015). Biochemical molecular analysis revealed that TIPE2 could reduce the activation of RAC1 and MMP9 by binding to RAC1, thereby suppressing gastric cancer cell metastasis (Xuelei et al., 2013). Also, TIPE2 is reported to elicit cell apoptosis by activating caspase three and caspase 9, inducing the cleavage of PARP, and inhibiting bcl-2 expression in BGC-823 gastric cancer cells. This information demonstrates that TIPE2 could be a novel and extremely potent gastric cancer target that suppresses gastric cancer through mediating multiple molecular pathways in patients with gastric tumors. However, it has not been well reported that several medicinal agents could regulate TIPE2 to fight against diseases such as gastric cancer. Therefore, the development of a TIPE2 regulator that suppresses gastric cancer and clarifying its function would be encouraging for the clinical treatment of gastric cancer and is urgently needed.

**FIGURE 9:**
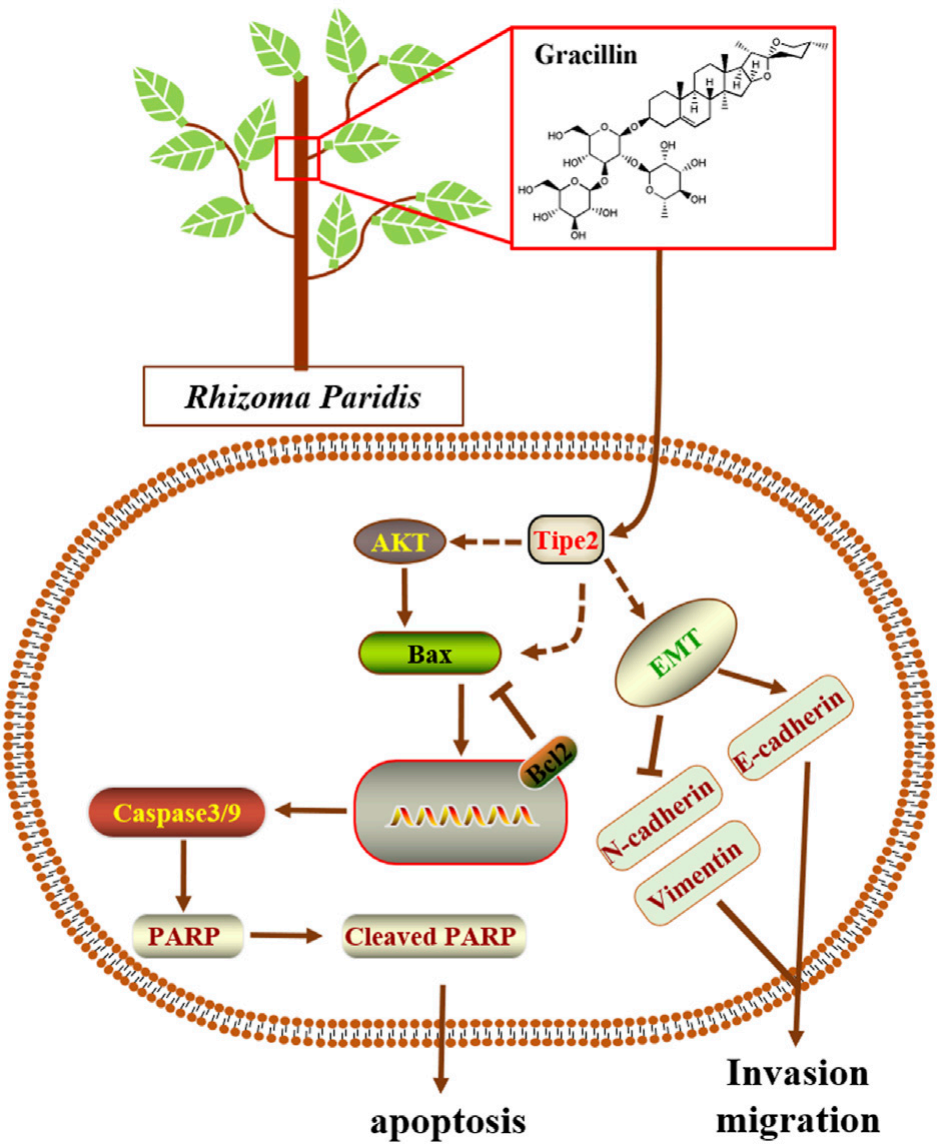
Scheme summarizing the mechanism of gracillin suppress gastric cancer via TIPE2.

Natural products are generally complex multi-ingredient systems and contain a great deal of different and remarkably similar structural compounds that have contributed to the majority of modern medicinal agents. However, these natural compounds were seldom reported to mediate TIPE2 and explore its medicinal value. In the present study, we employed four traditional natural plants with anti-tumor effects, respectively *Curcuma longa L., Tripterygium Wilfordii, Rhizoma Paridis,* and *Reynoutria japonica Houtt*, to screen the TIPE2 regulator fighting against gastric cancer in BGC823 cells, and found that the ethyl acetate extraction of *Rhizoma Paridis* exerted the induction of TIPE2 expression and inhibition of cellular proliferation. *Rhizoma Paridis* is a plant that generally grows in southwest China, and has been widely applied to prevent and treat chronic diseases including cancer as a traditional Chinese medicine with heat-clearing and detoxifying properties. Its numerous active ingredients have also been reported to exert multiple anti-cancer effects, eg. pennogenyl saponins for inhibiting the growth of hepatoma, dioscin for suppressing osteosarcoma via inducing cell cycle arrest and apoptosis, and polyphyllin II for relieving bladder cancer migration. However, the underlying anti-cancer mechanism of these active ingredients from *Rhizoma Paridis* remains unclear. In this context, we analyzed the active compounds based on bioassay-guided isolation combining with the reported ingredient information of *Rhizoma Paridis* and evaluated the underlying target and mechanism of the active compound fighting against gastric cancer. We identified gracillin as a TIPE2 inducer from *Rhizoma Paridis* and elucidated the potential mechanism by which gracillin alleviates gastric carcinoma via TIPE2-mediated inhibition of proliferation, induction of endogenous apoptosis, and suppression of migration (Figure 9). Our findings laid a theoretical understanding of the underlying mechanism and function of *Rhizoma Paridis* and provided an important insight for the modern medicinal development of *Rhizoma Paridis.*

Sustained proliferation is considered as a primary characteristic of malignant tumor cells differing from the restricted proliferation of normal cells. AKT, also named protein kinase B (PKB), is an important part of the PI3K/AKT/mTOR pathway and its phosphorylation is a sign of the pathway activation which exerts a vital effect on cell proliferation, including of malignant cells. Our previous findings revealed that TIPE2 expression was negatively associated with gastric cell proliferation, and that it could suppress cell proliferation by reducing phosphorylation of AKT. In the present study, we found that gracillin inhibited AKT phosphorylation and cell proliferation in gastric cancer BGC-823 cells, and in the absence of TIPE2, gracillin exhibited a more obvious induction of TIPE2 expression and inhibition of AKT phosphorylation, demonstrating that gracillin maybe inhibit the gastric cancer cells via TIPE2-mediated AKT phosphorylation. Additionally, cell cycle progression on the rails is recognized to be crucial to maintaining normal cell proliferation, and its dysfunction and un-limitation contribute to the development of malignant tumor cells. Our previous data revealed that TIPE2 overexpression could cause G2/M phase cell cycle arrest and regulate the expression of CDK1, phosphorylated CDK1, cyclin B1, and p21, and in the present study, gracillin also displayed a similar effect on cell cycle with TIPE2 overexpression. Together, these results confirmed that gracillin could inhibit gastric cancer cell proliferation via AKT phosphorylation and cell cycle distribution, which may be associated with TIPE2 expression.

Tumor metastasis is a typical and fatal symptom of advanced cancer, which contributes to approximately 90% of cancer-related deaths. During the process of tumor metastasis, malignant tumor cells begin to migrate via the epithelial-mesenchymal transition process (EMT), spread to the patient’s organs, and finally threaten the patient’s life. The EMT process is usually characterized by high expression of N-cadherin and vimentin and low expression of E-cadherin. Recent evidence has revealed that TIPE2 could suppress the migration of tumor cells by promoting the induction of E-cadherin expression and inhibition of N-cadherin and vimentin expression. Our present data show that gracillin suppresses the migration of gastric cancer BGC-823 cells, and that TIPE2 partly participated in this effect. Furthermore, gracillin induced the expression of E-cadherin and inhibited the expression of N-cadherin and vimentin, and cells treated with TIPE2 siRNA displayed a more obvious regulation effect of gracillin on E-cadherin and vimentin expression. These results reveal that gracillin possesses the potential to suppress tumor cell migration via the EMT process in BGC-823 cells, which may be associated with TIPE2 expression.

Additionally, cell apoptosis is recognized as a process of programmed cell death that maintains physiological homeostasis by clearing away the dysfunctional and valueless cells. However, the resistance to apoptosis is a classic characteristic of malignant tumor development. Hence, reducing the resistance to apoptosis would be an important strategy for fighting tumors. Bcl2, an antiapoptotic protein found in mitochondria, plays an important role in determining the occurrence of apoptosis and regulating tumor progression. Several previous reports revealed that TIPE2 could inhibit the expression of bcl2 and suppress mitochondrial apoptosis as a tumor suppressor in gastric cancer cells and other tumor cells. This information implied that the effects of gracillin fighting against gastric cancer were also associated with TIPE2-mediation of mitochondrial apoptosis. We investigated this issue and confirmed that gracillin could promote cell apoptosis and suppress the expression of bcl2, and that the effect of gracillin was statistically more significant in the absence of TIPE2. These results demonstrated that TIPE2-mediation of mitochondrial apoptosis may also be a way that gracillin suppresses gastric cancer. Of course, the role of mitochondria needs to be elucidated in future research, to better understand the pro-apoptotic effect of gracillin.

Overall, the present study has identified gracillin as a TIPE2 inducer that can fight against gastric carcinoma, which was extracted from the medicinal plant *Rhizoma Paridis* following bioassay-guided isolation. Furthermore, we elucidated the mechanism of gracillin’s benefits against gastric carcinoma: that gracillin inhibits cell proliferation involving the PI3K/AKT pathway and cell cycle arrest, suppresses the EMT pathway to regulate cell migration, and induces bcl2-associated mitochondrial apoptosis. Moreover, we confirmed the role of TIPE2 in these effects. These findings regarding the benefits and mechanism of gracillin isolated from *Rhizoma Paridis* will be beneficial to the modern medicinal development of *Rhizoma Paridis* and gracillin, and it is also interesting to understand the role of TIPE2 in gastric carcinoma. Of course, several issues regarding how gracillin regulates TIPE2 and the more precise mechanism of TIPE2 in gastric carcinoma remain obscure. Our laboratory will continue to explore these issues, and some of this work is already underway.

## Data Availability

The raw data supporting the conclusions of this article will be made available by the authors, without undue reservation.
