# Supplementary figures and images for: Beneficial Effects of Gracillin From Rhizoma Paridis Against Gastric Carcinoma via the Potential TIPE2-Mediated Induction of Endogenous Apoptosis and Inhibition of Migration in BGC823 Cells
Source: Front Pharmacol. 2021 Sep 24;12:669199. doi: 10.3389/fphar.2021.669199 (PMC8497801; doi:10.3389/fphar.2021.669199)

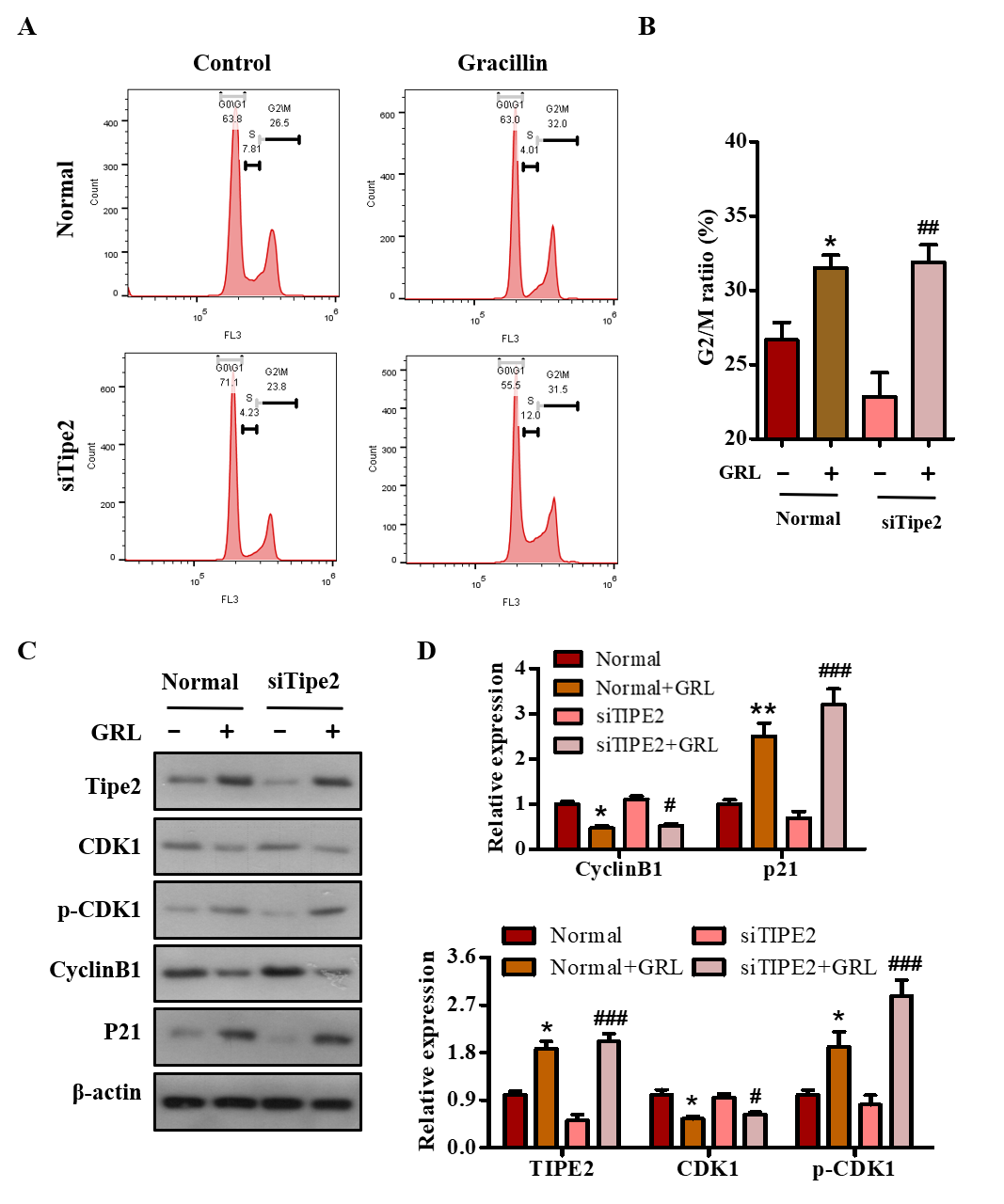

Supplement: Supplementary file 1 [file Figure5.TIF]

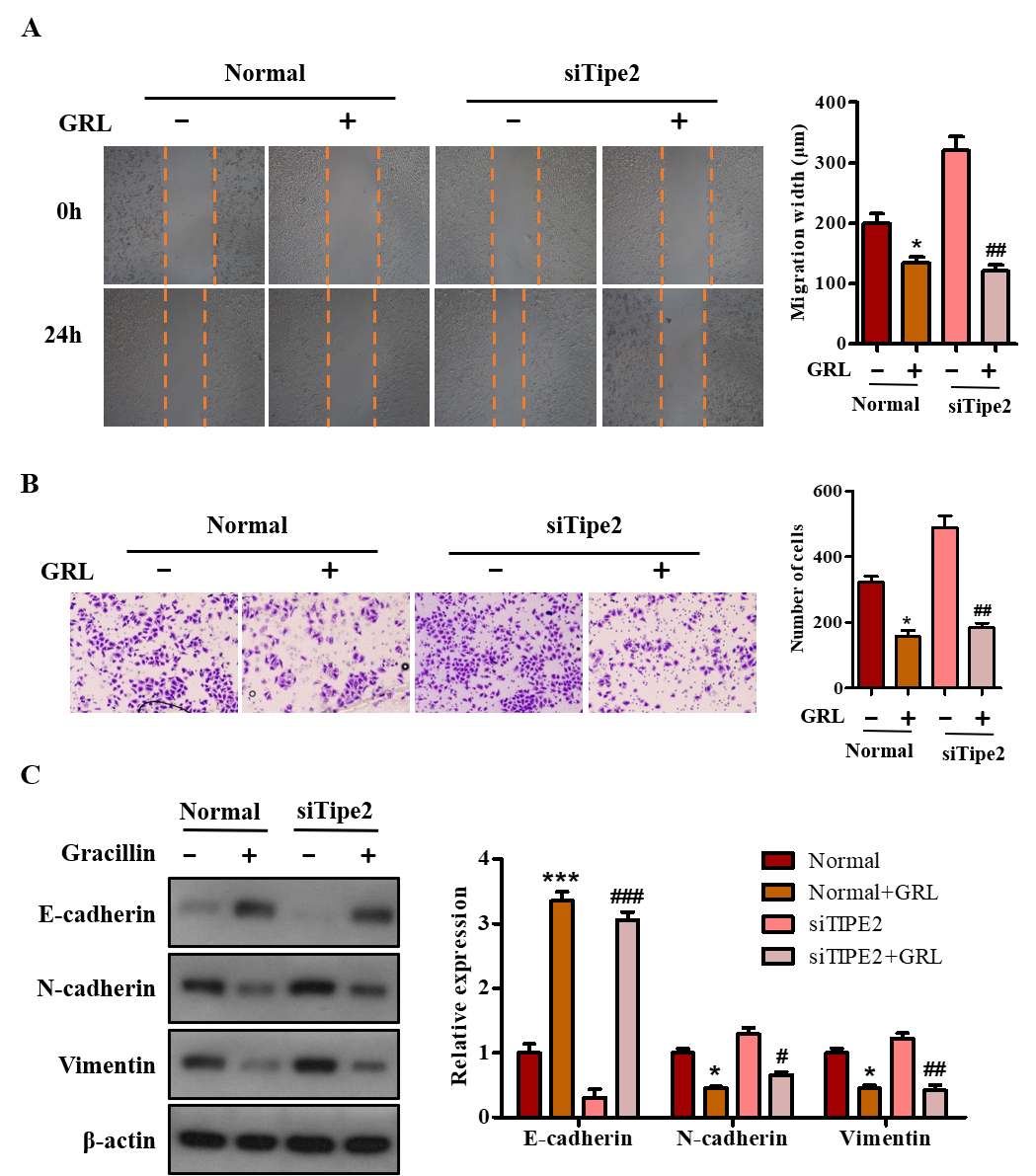

Supplement: Supplementary file 2 [file Figure6.TIF]

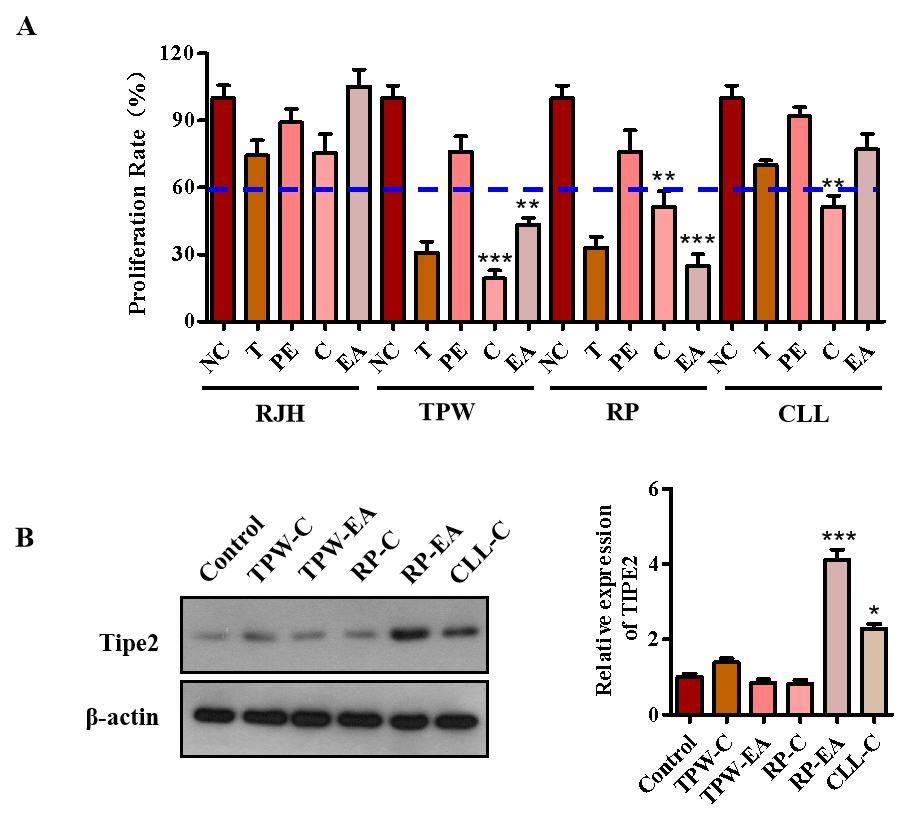

Supplement: Supplementary file 4 [file Figure1.TIF]

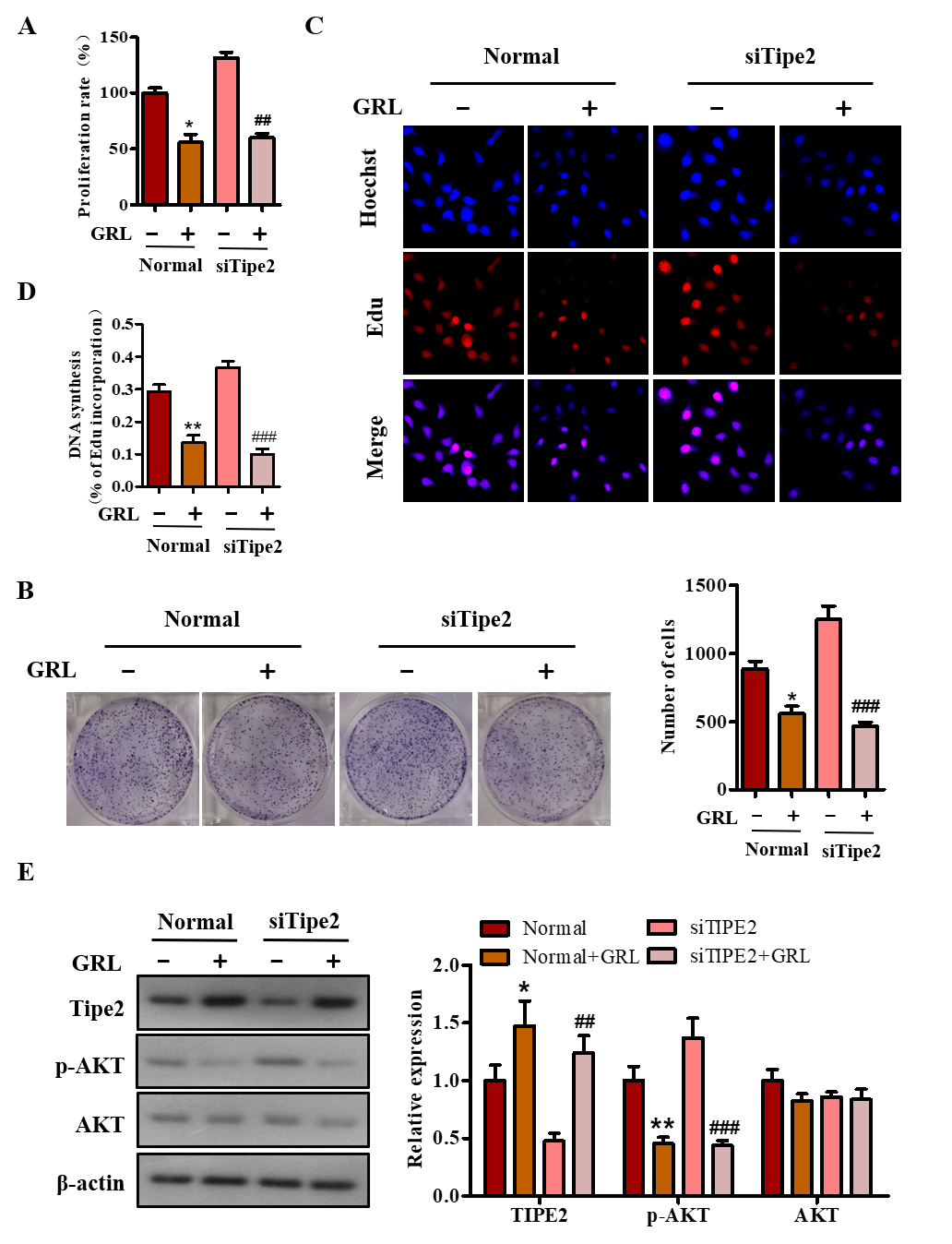

Supplement: Supplementary file 5 [file Figure4.TIF]

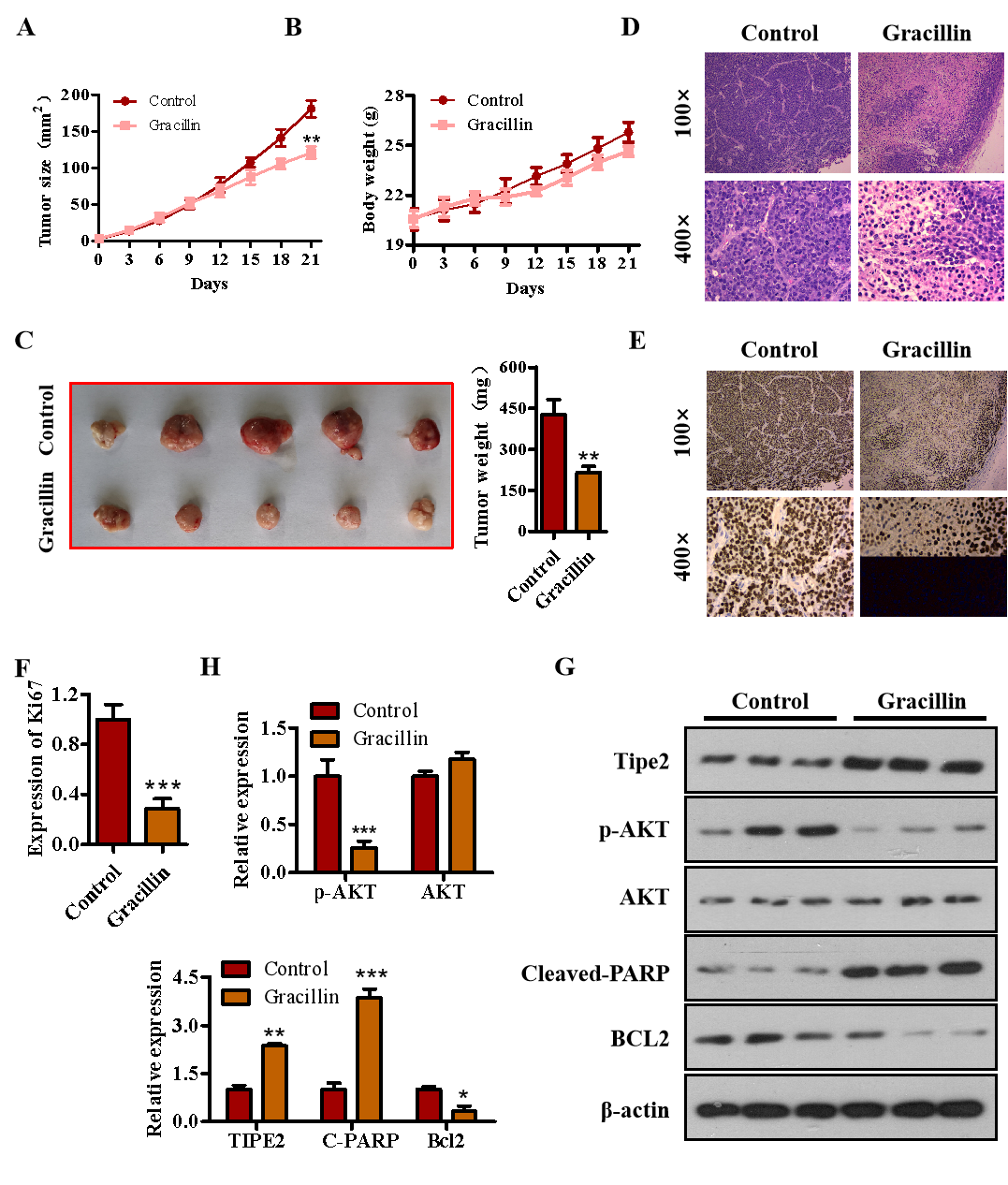

Supplement: Supplementary file 6 [file Figure8.TIF]

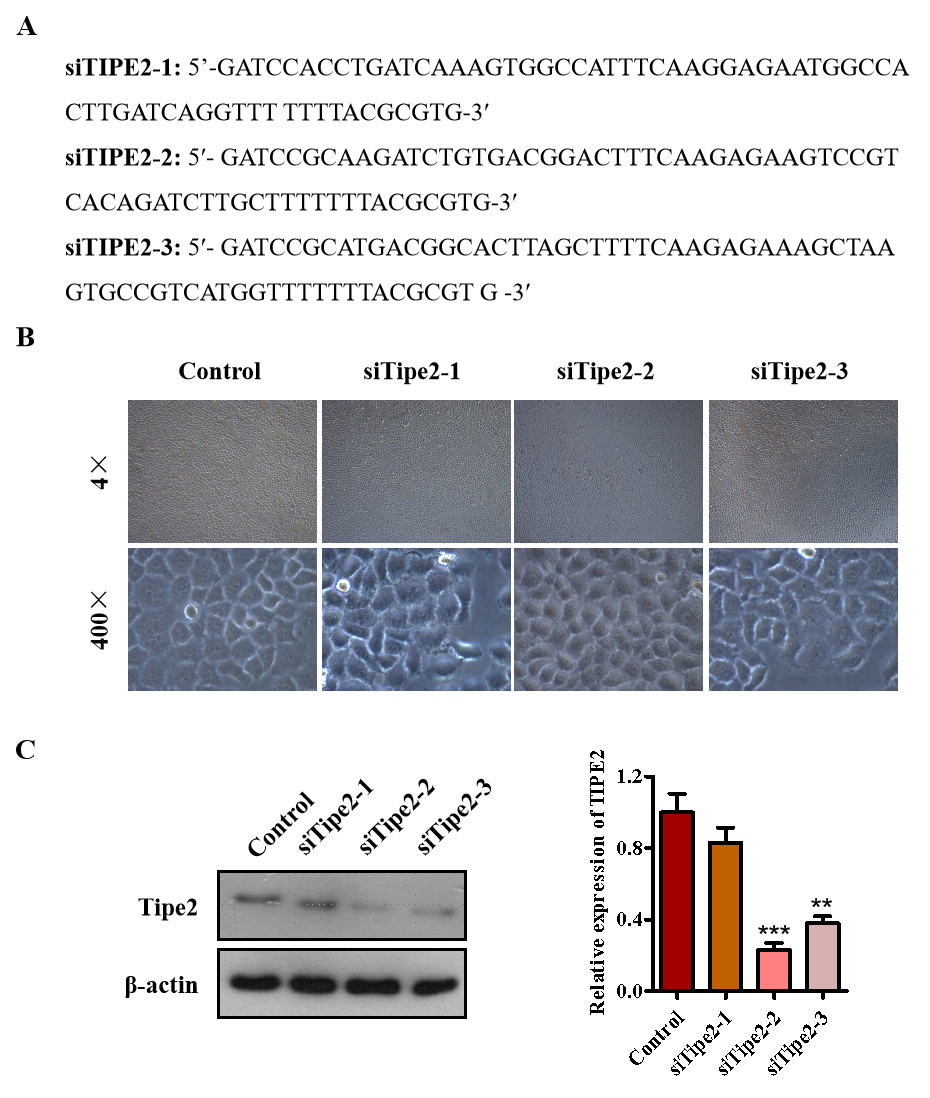

Supplement: Supplementary file 8 [file Figure3.TIF]

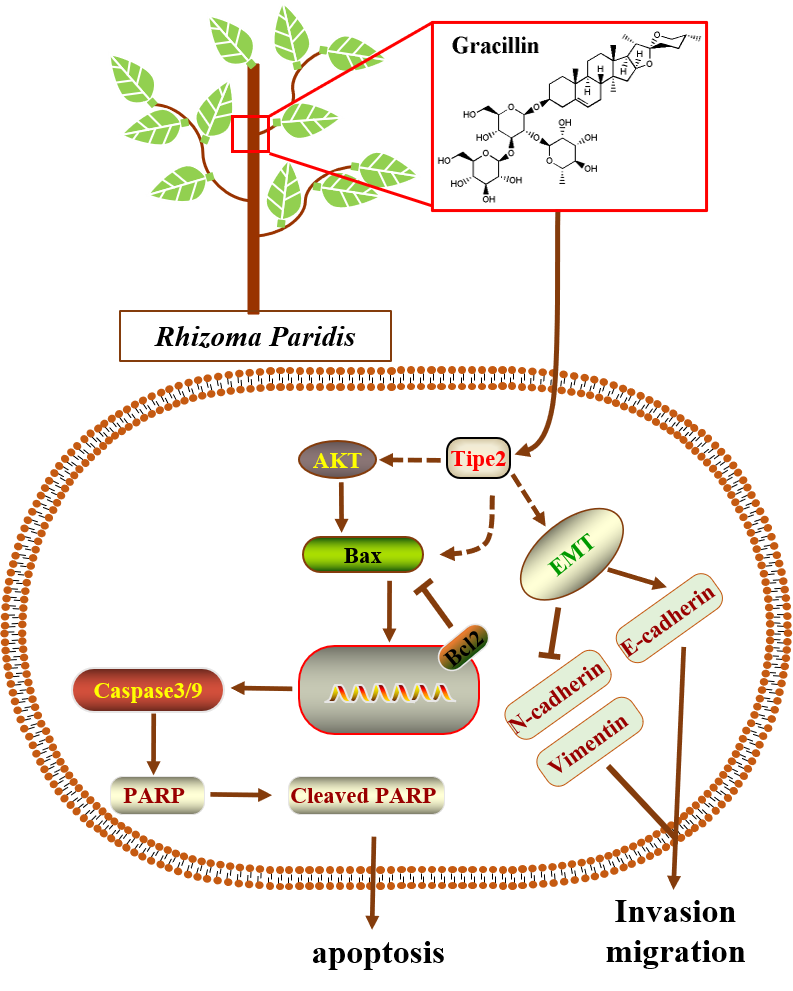

Supplement: Supplementary file 9 [file Figure9.TIF]

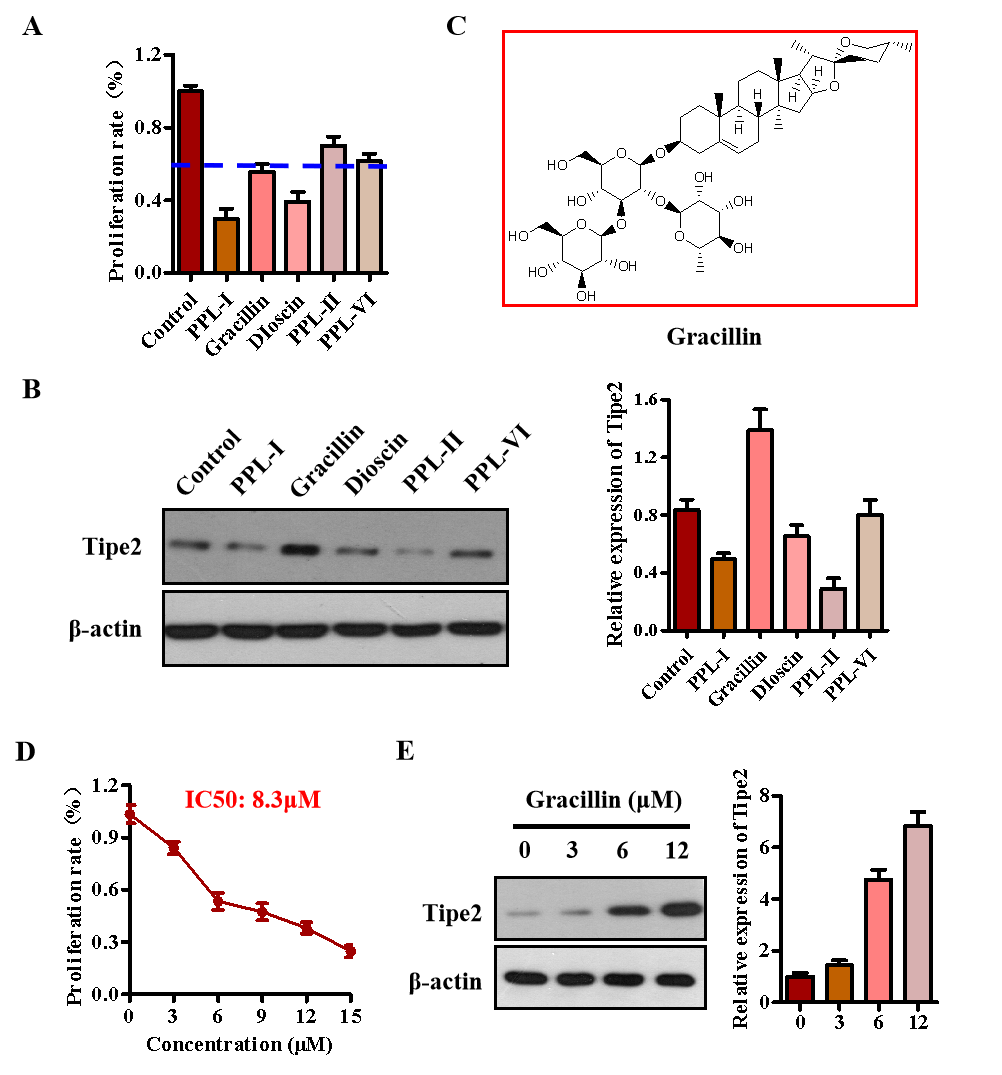

Supplement: Supplementary file 10 [file Figure2.TIF]

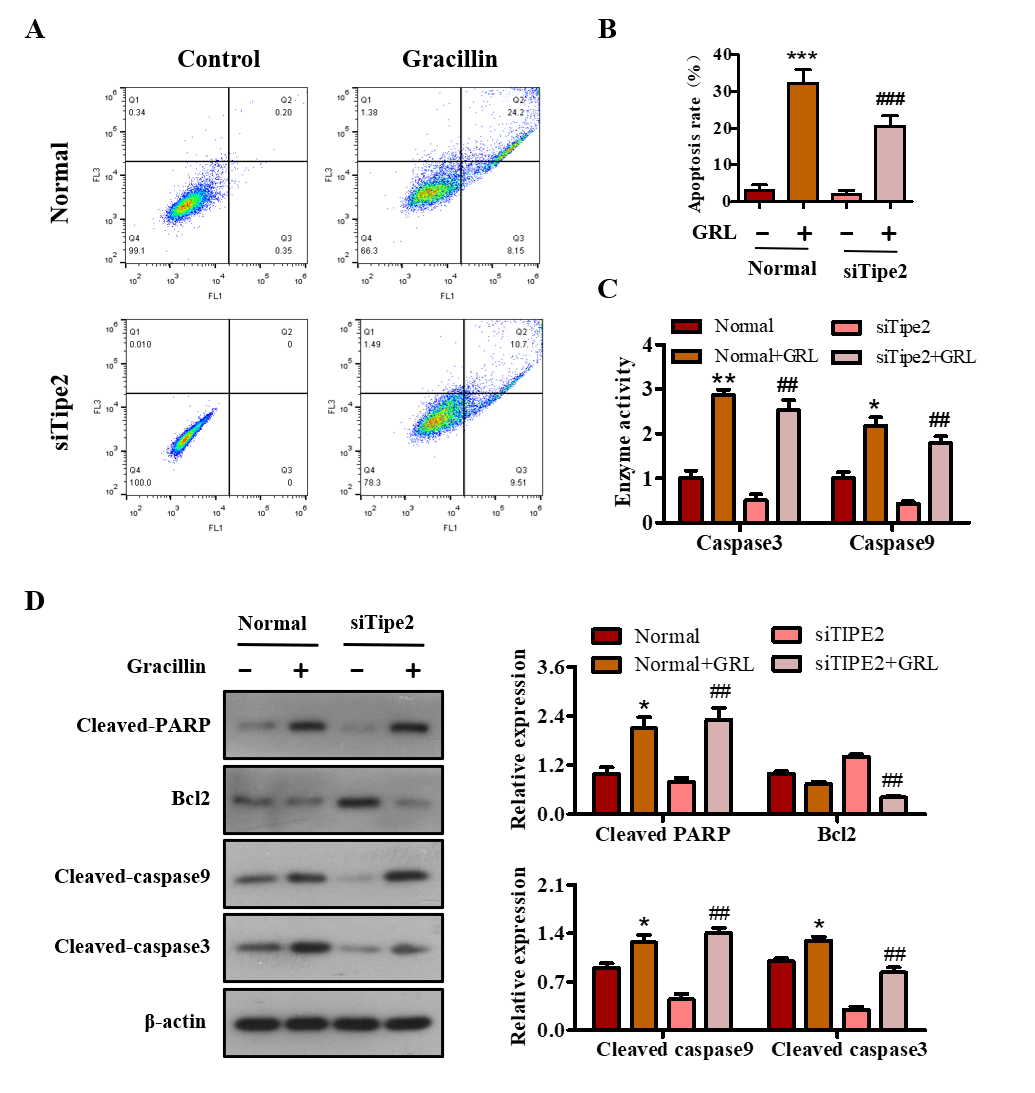

Supplement: Supplementary file 11 [file Figure7.TIF]
