# Supplementary material for: Beneficial Effects of Gracillin From Rhizoma Paridis Against Gastric Carcinoma via the Potential TIPE2-Mediated Induction of Endogenous Apoptosis and Inhibition of Migration in BGC823 Cells
Source: Front Pharmacol. 2021 Sep 24;12:669199. doi: 10.3389/fphar.2021.669199 (PMC8497801; doi:10.3389/fphar.2021.669199)
Supplement: Supplementary file 3 [file Presentation1.pdf]

Supplemental Information

Beneficial effects of Gracillin from *Rhizoma Paridis* against gastric carcinoma via TIPE2-mediated induction of endogenous apoptosis and inhibition of migration in BGC832 cells

Wenming Liu[1, #], Yanting Wang[1, #], Junjie Chen[1], Zhenhe Lin[1], Mengjie Lin[2], Xiantong Lin[3, *], Yanyun Fan[1, *]

[1]Department of Gastroenterology, Zhongshan Hospital, Xiamen University, Xiamen 361004, Fujian Province, China

[2]Department of Pathology, Zhongshan Hospital, Xiamen University, Xiamen 361004, Fujian Province, China

[3]Department of Thoracic and Cardiovascular Surgery, The Third Hospital of Xiamen

*Correspondence: Yanyun Fan, trudy1@163.com

[#]The authors contributed equally.

Figure S1

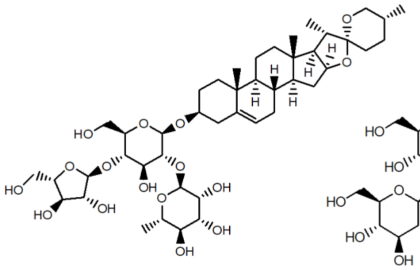

Polyphyllin I

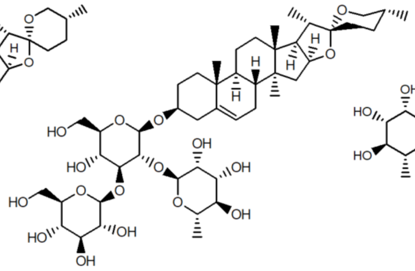

Gracillin

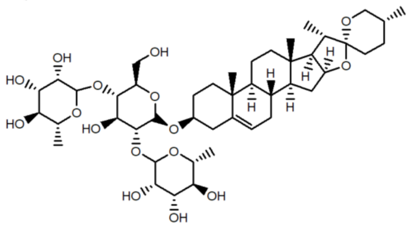

Dioscin

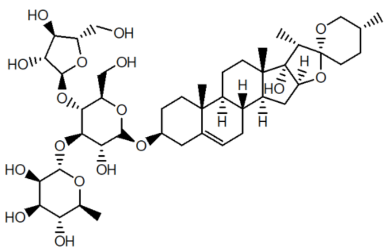

Polyphyllin II

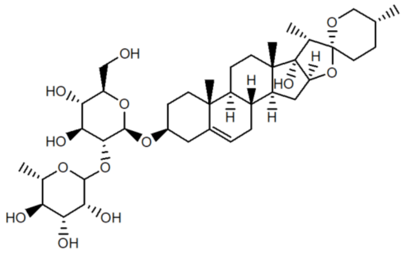

Polyphyllin VI

Figure S1 The structures of five active compounds

Figure S2

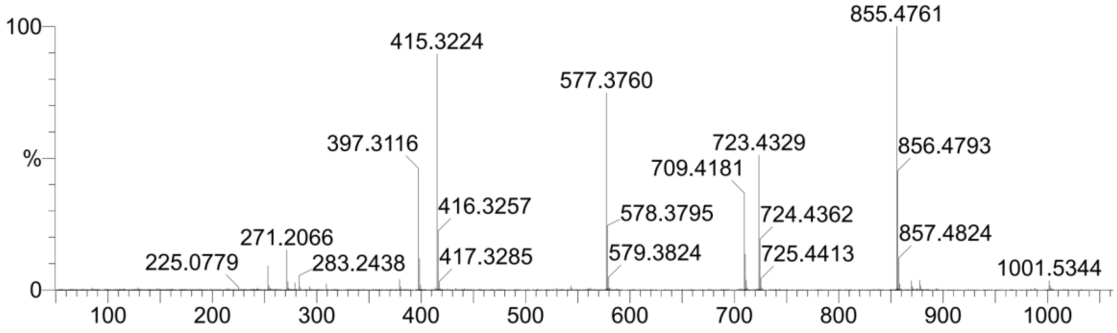

Figure S2 The MS spectra of polyphyllin I

Figure S3

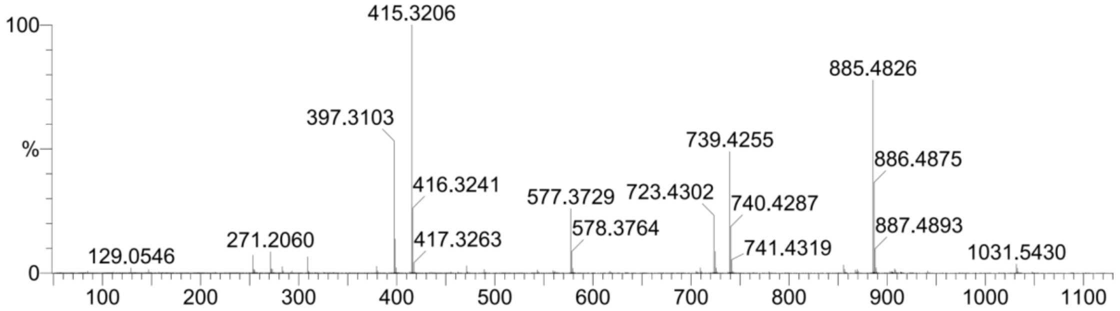

Figure S3 The MS spectra of gracillin

Figure S4

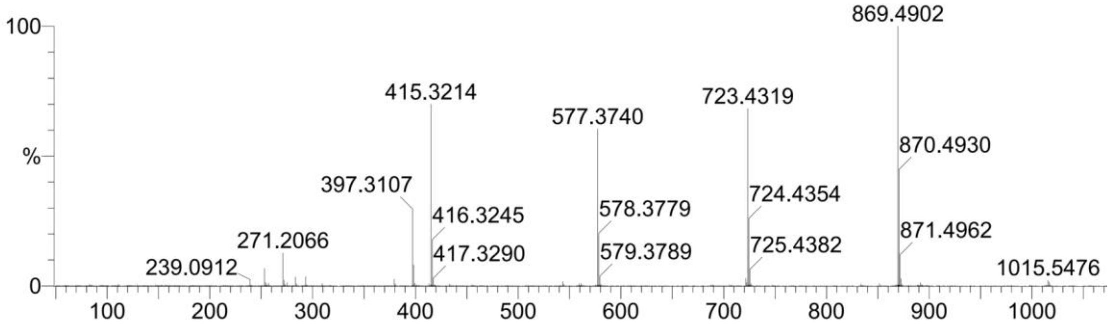

Figure S4 The MS spectra of dioscin

Figure S5

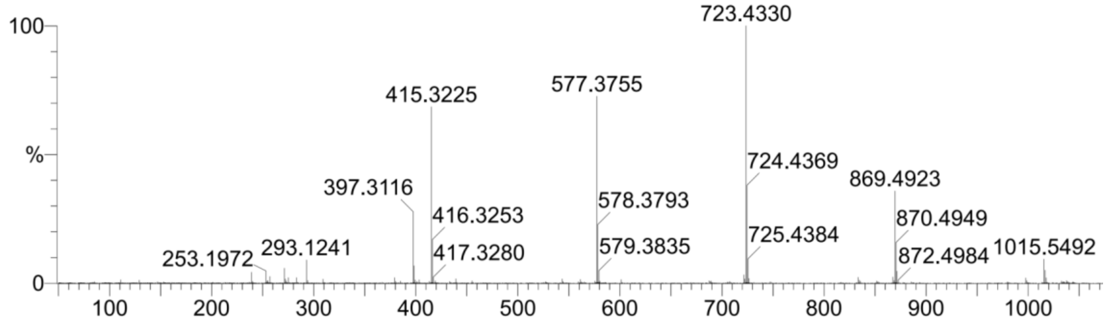

Figure S5 The MS spectra of polyphyllin II

Figure S6

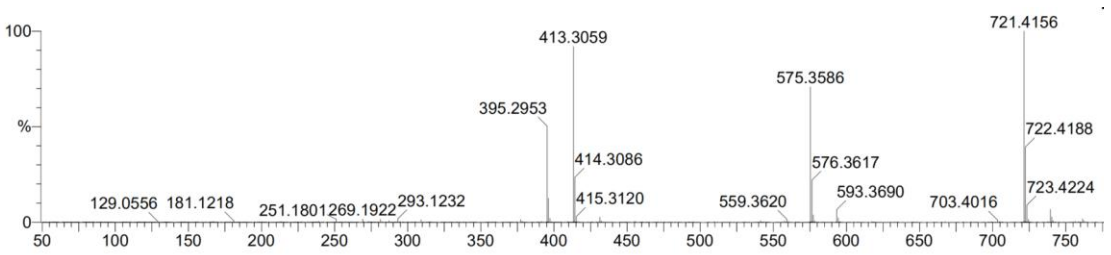

Figure S6 The MS spectra of polyphyllin VI

Figure S7

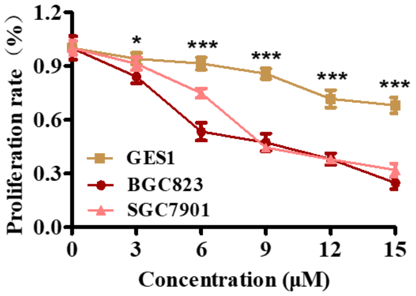

Figure S7 The effects of gracillin with the different concentrations on cell proliferation in normal gastric cell line GES1, gastric cancer cell lines BGC823 and SGC7901

Figure S8

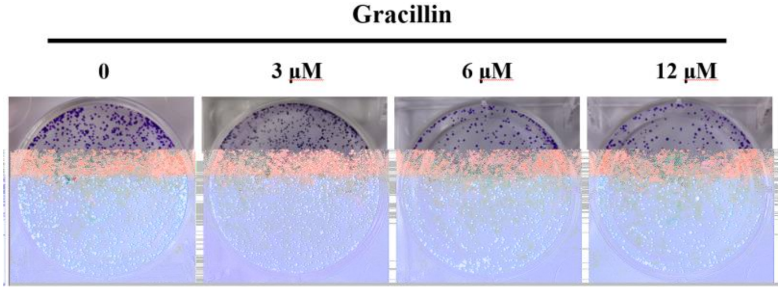

Figure S8 Colon formation for cell proliferation by gracillin in gastric cancer cell line SGC7901.

Figure S9

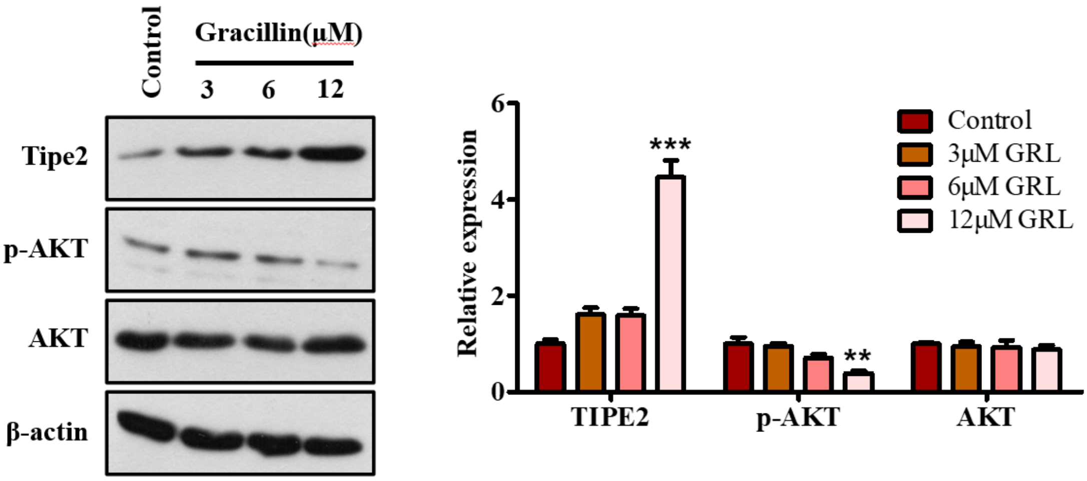

Figure S9 Western blot for detecting the protein expression of TIPE2, AKT and p-AKT mediated by gracillin in gastric cancer cell line SGC7901.

Figure S10

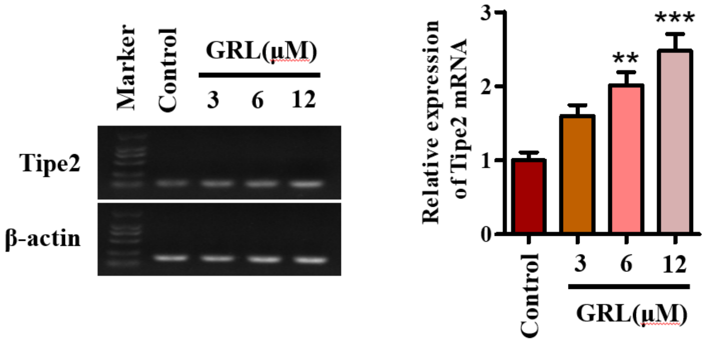

Figure S10 RT-PCR for detecting the mRNA expression of TIPE2 mediated by gracillin in gastric cancer cell line BGC823.

Figure S11

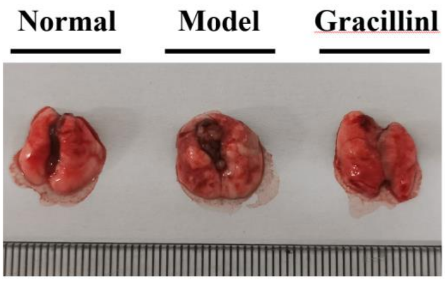

Figure S11 The representative images from lung of mice for lung metastases.

Figure S12

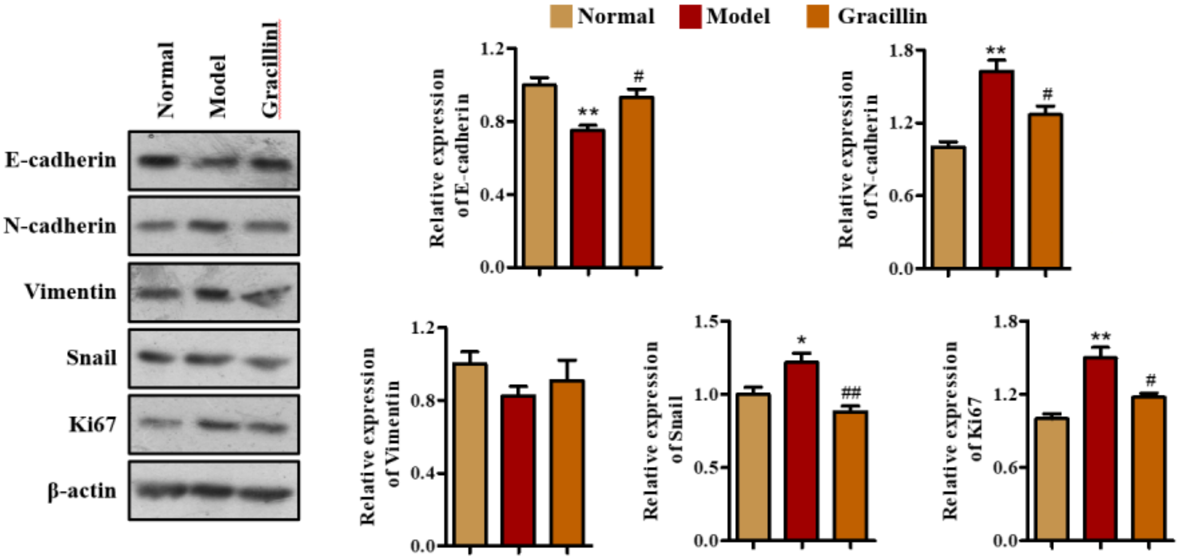

Figure S12 Western blot for detecting the tumor proliferation and metastases-associated protein expressions of ki67, E-cadherin, N-cadherin, vimentin and snail in lung tissue of mice.
