# Supplementary material for: Beneficial Effects of Gracillin From Rhizoma Paridis Against Gastric Carcinoma via the Potential TIPE2-Mediated Induction of Endogenous Apoptosis and Inhibition of Migration in BGC823 Cells
Source: Front Pharmacol. 2021 Sep 24;12:669199. doi: 10.3389/fphar.2021.669199 (PMC8497801; doi:10.3389/fphar.2021.669199)
Supplement: Supplementary file 7 [file DataSheet1.zip › Table 1.DOCX]

**Supplemental Information**

**Beneficial effects of Gracillin from** ***Rhizoma Paridis* against gastric carcinoma via TIPE2-mediated induction of endogenous apoptosis and inhibition of migration in BGC832 cells**

Wenming Liu^1, #^, Yanting Wang^1, #^, Junjie Chen^1^, Zhenhe Lin^1^, Mengjie Lin^2^, Xiantong Lin^3, *^, Yanyun Fan^1, *^

^1^Department of Gastroenterology, Zhongshan Hospital, Xiamen University, Xiamen 361004, Fujian Province, China

^2^Department of Pathology, Zhongshan Hospital, Xiamen University, Xiamen 361004, Fujian Province, China

### ^3^[Department of Thoracic and Cardiovascular Surgery](http://citeseerx.ist.psu.edu/viewdoc/summary?doi=10.1.1.289.5424), The Third Hospital of Xiamen

*Correspondence: Yanyun Fan, [trudy1@163.com](mailto:trudy1@163.com)

^#^The authors contributed equally.

**Figure S1**


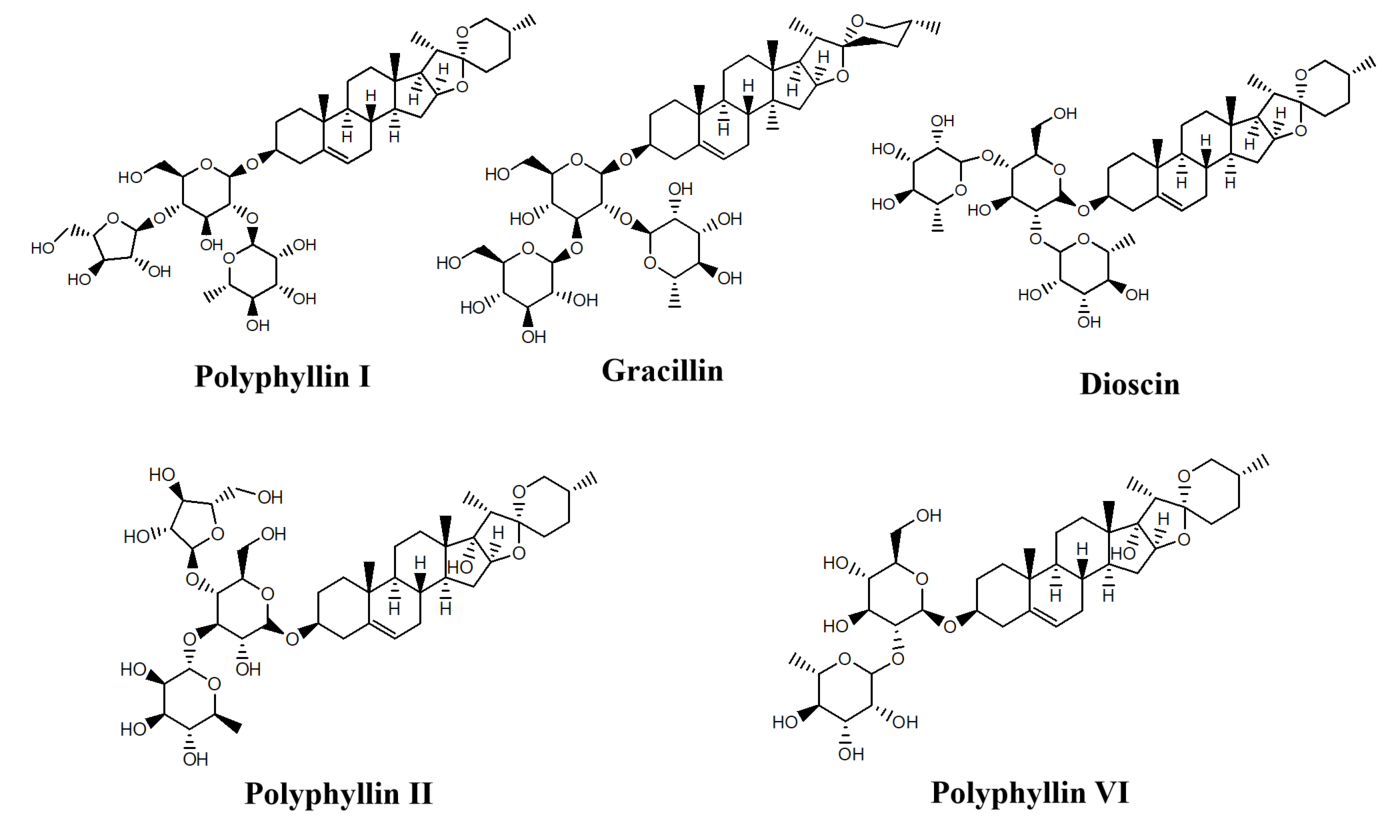


Figure S1 The structures of five active compounds

**Figure S2**


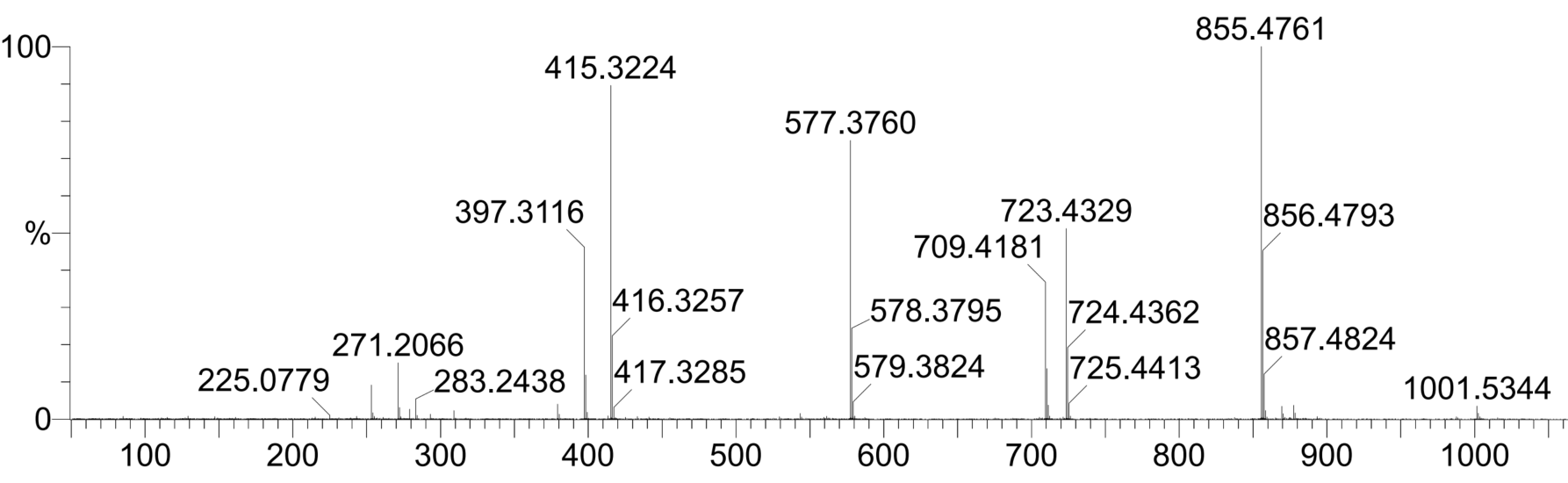


Figure S2 The MS spectra of polyphyllin I

**Figure S3**


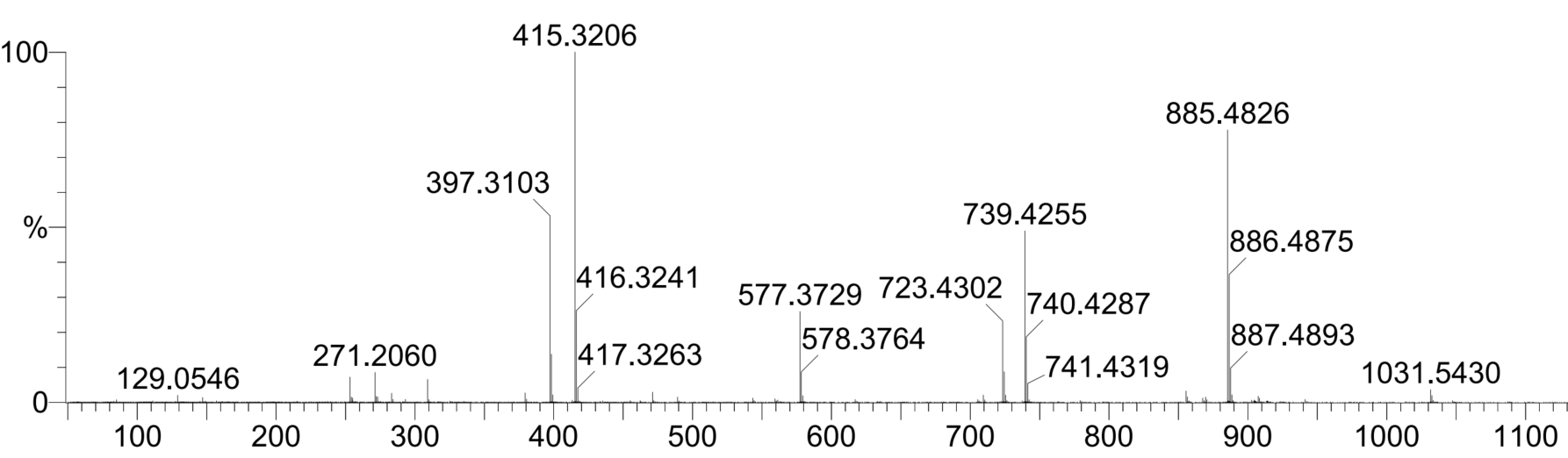


Figure S3 The MS spectra of gracillin

**Figure S4**


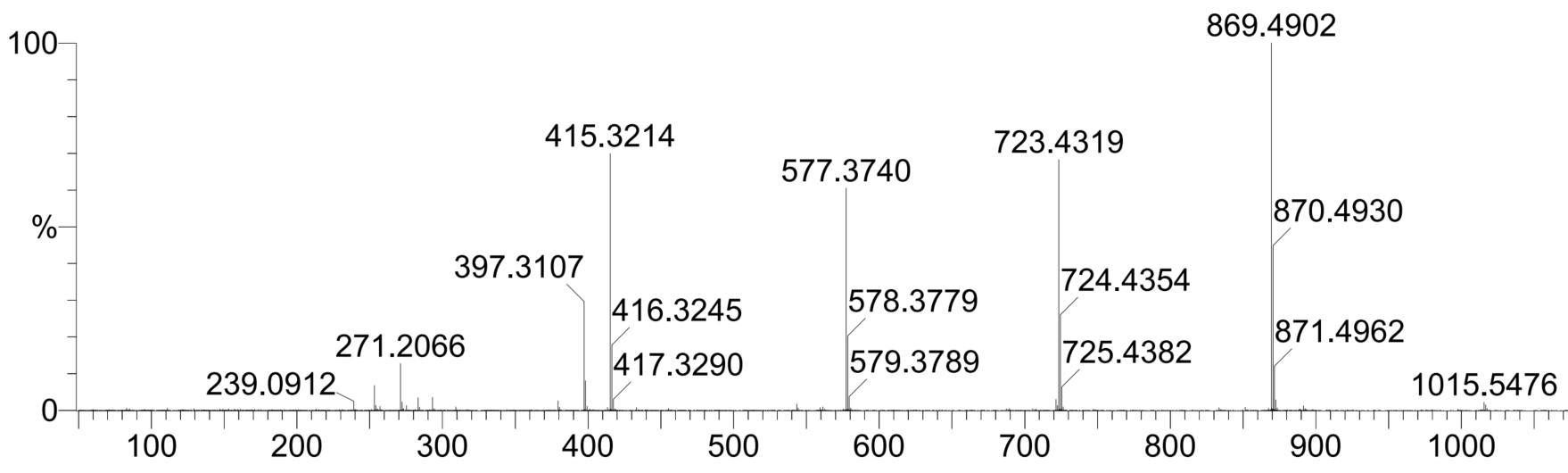


Figure S4 The MS spectra of dioscin

**Figure S5**


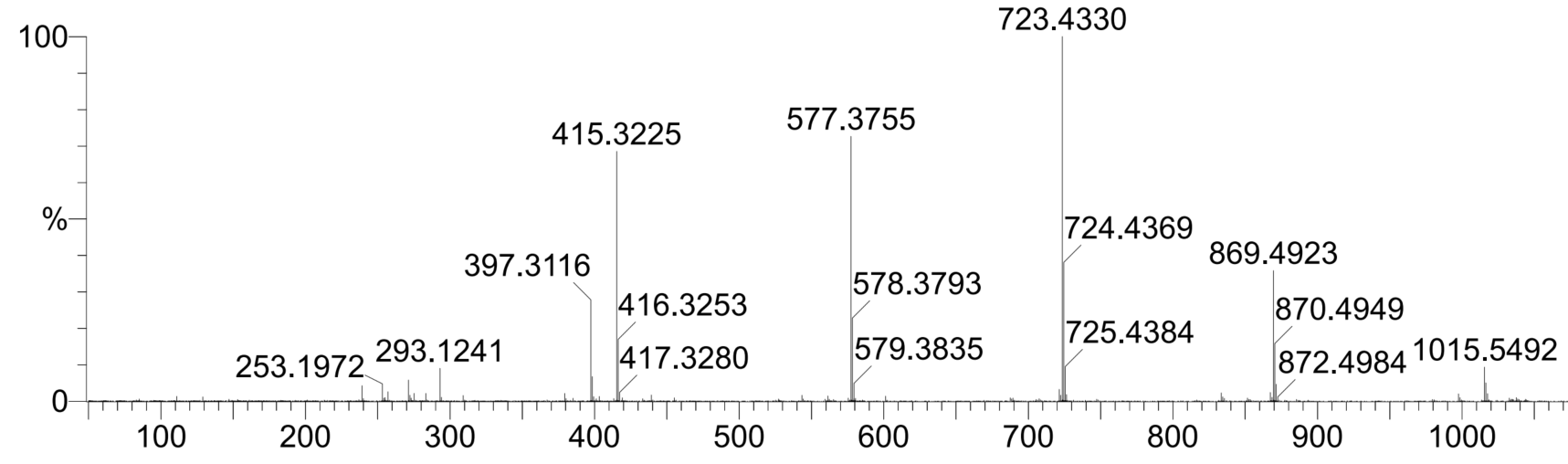


Figure S5 The MS spectra of polyphyllin II

**Figure S6**


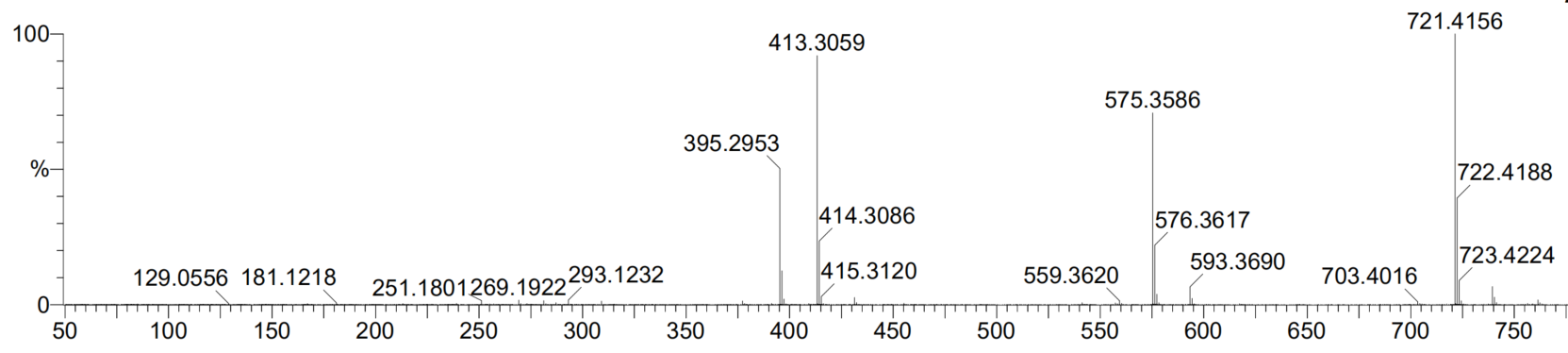


Figure S6 The MS spectra of polyphyllin VI

**Figure S7**

**
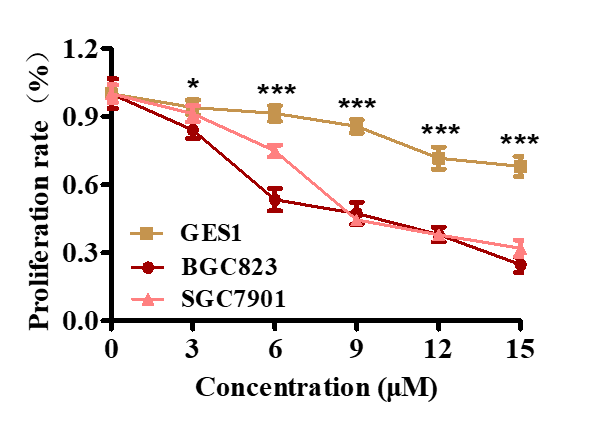
**

Figure S7 The effects of gracillin with the different concentrations on cell proliferation in normal gastric cell line GES1, gastric cancer cell lines BGC823 and SGC7901

**Figure S8**


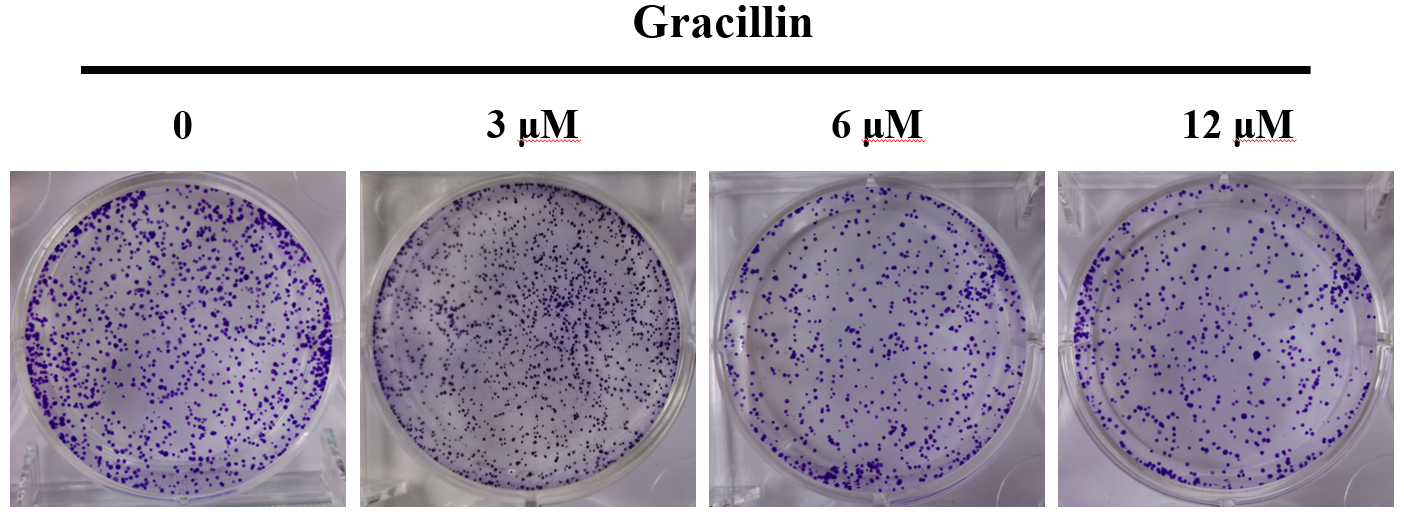


Figure S8 Colon formation for cell proliferation by gracillin in gastric cancer cell line SGC7901.

**Figure S9**


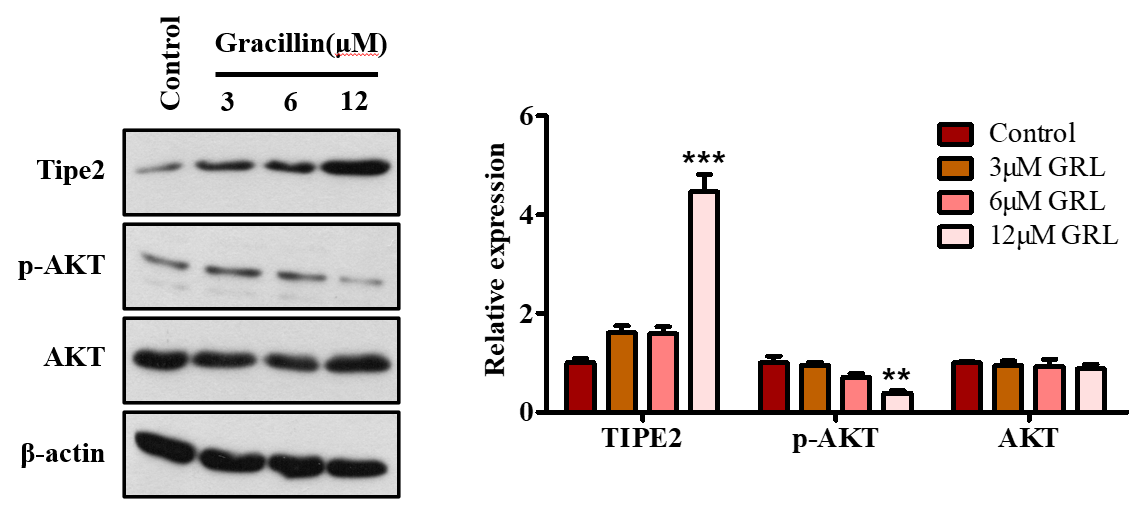


Figure S9 Western blot for detecting the protein expression of TIPE2, AKT and p-AKT mediated by gracillin in gastric cancer cell line SGC7901.

**Figure S10**


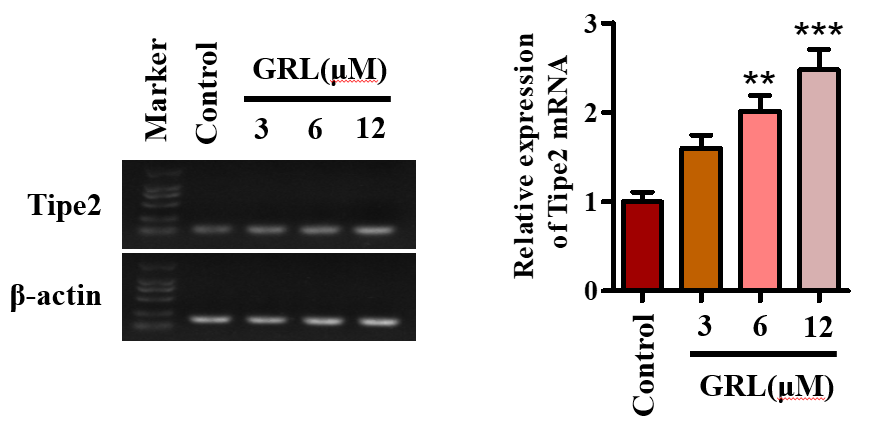


Figure S10 RT-PCR for detecting the mRNA expression of TIPE2 mediated by gracillin in gastric cancer cell line BGC823.
